# Mechanochemical coupling in lung cancer: how tissue tension drives genomic instability and malignant progression

**DOI:** 10.3389/fcell.2026.1936991

**Published:** 2026-10-20

**Authors:** Tianli Wang, Qiang Xiao

**Affiliations:** Department of Pulmonary and Critical Care Medicine, Changde Hospital, Xiangya School of Medicine, Central South University (The First People’s Hospital of Changde City), Changde, Hunan, China

**Keywords:** genomic instability, lung cancer, mechanochemical coupling, mechanopharmacology, oxidative stress, tumor microenvironment

## Abstract

Recent studies have called into question the classical “genocentric” view of lung carcinogenesis, positing instead that the tumor physical microenvironment is a major contributor to malignant progression. This review synthesizes the current evidence to propose a mechanochemical coupling framework, in which pathological mechanical forces, specifically high-magnitude cyclic stretch and matrix stiffening, are hypothesized to be converted into sustained oxidative stress and genomic instability within the lung tumor microenvironment. We describe a tiered activation mechanism by which mechanical forces (via Piezo1 and integrin-Rac1 pathways) may trigger an initial burst of reactive oxygen species (ROS), which then leads to mitochondrial fragmentation by the action of dynamin-related protein 1 (DRP1) and ROS-induced ROS generation. At the same time, mechanical dissociation of p120-catenin from adherens junctions (AJs) may activate Toll-like receptor 4 (TLR4)/nuclear factor kappa B (NF-κB) signaling and further amplify pro-oxidative feedforward mechanisms, while pathological tension may promote endothelial nitric oxide synthase (eNOS) uncoupling and tetrahydrobiopterin (BH4) depletion, further exacerbating the oxidative burden. At the nuclear level, transmission of mechanical signals downstream of the linker of nucleoskeleton and cytoskeleton (LINC) complex and Lamin A/C network may lead to chromatin opening, altered nuclear pore integrity, accumulation of oxidative damage (8-OHdG), and suppression of repair pathways (Akt/Nrf2), potentially contributing to mutational fixation. These mechanochemical insults may induce epithelial–mesenchymal transition (EMT), activation of the NOD-like receptor family pyrin domain-containing 3 (NLRP3) inflammasome, high-mobility group box 1 (HMGB1)-driven inflammatory signaling, and remodeling of an immune-evasive niche. Finally, we propose mechanopharmacology as a theoretical dual biochemical–physical approach to intercept malignancy at its mechanical roots, shifting from the mutation-driven paradigm toward system physics oncology.

## 1 Introduction

The dominant view of the initiation and progression of lung cancer over the past few decades has been “genocentric” based on the random acquisition of genetic mutations and epigenetic changes. This view does not always account for the strong influence that the physical microenvironment (“mechanical niche”) has on neoplastic or pre-neoplastic cells (Gong et al., 2025). The lung, as a dynamic organ, undergoes continuous rhythmic deformation and is subject to complex mechanical stresses during physiological function. Growing evidence indicates that the development of lung cancer is not only a biochemical signaling defect but also a potential failure of mechanical homeostasis (Wang and Yang, 2022). This paradigm shift suggests that mechanical forces may play a key role in the malignant evolution of lung cancer, rather than being a mere by-product of tissue remodeling (Butcher et al., 2009). There is a current consensus that this conceptual framework is a plausible interpretation of lung tumorigenesis, although quantitative data supporting it still need to be further supplemented.

At the heart of this mechanical-to-biological transition is mechanochemical coupling. This theoretical mechanism outlines how physical forces, including solid stress from tumor expansion, matrix stiffness associated with fibrosis, and cyclic stretch from disorganized ventilation, may be translated across scales into pro-destructive biochemical signals (Birukov, 2009; Chatterjee and Fisher, 2014). In the lung microenvironment, abnormal tensile strain acts as a physical stimulus that may be “coupled” to intracellular production of reactive oxygen species (ROS) and subsequent DNA damage. Specifically, high-magnitude cyclic stretch (e.g., ∼20% strain in experimental 2D elastomeric membrane models) triggers an explosive oxidative burst, which is primarily induced by the activation of NADPH oxidase 2 (NOX2) and mitochondrial dysfunction *in vitro* (Chapman et al., 2005; Browning et al., 2012). It is critical to note that this ∼20% threshold represents an experimental upper-bound strain model commonly employed to unmask mechanosensitive pathways, rather than a direct mirror of routine *in vivo* tumor-associated strain, which is buffered by the three-dimensional (3D), viscoelastic, and stress-relaxing properties of native lung parenchyma. Accumulating *in vitro* evidence supports the concept that mechanochemically induced oxidative stress may serve as a bridge between the macro-scale physical environment and micro-scale genomic instability, consistent with the broader consensus that mechanical forces can drive pathways promoting tumor progression (Simi et al., 2015; van der Vliet et al., 2018).

In this context, the lung’s pathological mechanical environment may act as a “mechanochemical engine” that produces conditions favoring genomic instability. Chronic mechanical stress causes DNA double-strand breaks (DSBs) and may disrupt endogenous repair systems, including the protein kinase B (Akt)/nuclear factor erythroid 2-related factor 2 (Nrf2) pathway, leading to a state of persistent genomic fragility (Arimura et al., 2012; Zhang D. et al., 2024). However, direct deep-sequencing evidence demonstrating that mechanical tension alone generates permanent, fixed driver mutations (e.g., in EGFR, KRAS, or TP53) in intact 3D tissue remains a critical empirical gap. This coupling may also trigger a cascade of proinflammatory events, such as release of high-mobility group box 1 (HMGB1) and activation of the NOD-like receptor family pyrin domain-containing 3 (NLRP3) inflammasome, processes that may be further exacerbated under combined mechanical and oxidative stress (Sayan and Mossman, 2016; Wang M. et al., 2019). All these events may contribute to a vicious cycle of inflammation, tissue injury, and malignant transformation, linking genomic instability to tumorigenesis through well-documented associations (Arimura et al., 2012; Xian et al., 2022).

The goal of this review is to present an integrated, evidence-tiered overview of the proposed role of mechanochemical coupling in lung cancer evolution. We first describe the mechanical landscape of the lung tumor microenvironment (TME), focusing on matrix stiffness and pathological tension. We then discuss the molecular components underlying this hypothesized coupling, including mechanical NOX2 activation, mitochondrial fragmentation, and the potential role of p120-catenin in mitochondrial homeostasis. Finally, we examine the putative malignant consequences of these processes, such as epithelial–mesenchymal transition (EMT) and immune evasion, and introduce mechanopharmacology as a conceptual framework for lung cancer prevention and therapeutic intervention. Throughout this manuscript, we classify our findings into three evidence categories: established biophysical facts, cross-disciplinary extrapolations borrowed from other biological systems, and theoretical paradigms that still require validation in 3D or *in vivo* experimental settings. Drawing on principles from biomechanics and molecular oncology, this work offers a fresh perspective that lung cancer may represent a disorder of disrupted mechanical–chemical integration. This view is supported by current mechanobiological observations and awaits further clinical translation.

## 2 The biomechanical landscape of the lung tumor microenvironment

The functional integrity of the respiratory system depends on the delicate balance of physical forces, which are gradually disrupted during malignant transformation (Garcia et al., 2006; Shiraishi et al., 2023). This “mechanical dyshomeostasis” may switch the lung from a compliant physiological state to a mechanical environment of measurable stresses, with mechanical stiffness and strain potentially determining the cell fate (Broders-Bondon et al., 2018; Shiraishi et al., 2023).

### 2.1 Pathological forces: evolution of matrix stiffness and cyclic stretch

The lung is a highly dynamic organ, and its cells are constantly exposed to mechanical stimuli (Gong et al., 2021). When the lung cells become malignant, there may be a dramatic change in the mechanical environment from physiological elasticity to pathological rigidity and strain (Wang and Yang, 2022). This “biomechanical remodeling” may not merely be a consequence of tumor growth but could contribute to the development of a malignant phenotype, in line with a recent mechanobiological consensus that mechanical factors can regulate tumor behavior (Paszek et al., 2005; Mancini et al., 2024).

#### 2.1.1 Matrix stiffness: fibrotic rigidity as a malignant catalyst

An important hallmark of the lung cancer microenvironment is the excessive stiffness of the extracellular matrix (ECM) (Guo et al., 2022). The lung parenchyma is very compliant (typically Young’s modulus between 1 and 5 kPa) under normal physiologic conditions, permitting efficient gas exchange and elastic recoil (Chang et al., 2015). However, this landscape is further complicated by a “desmoplastic reaction,” mediated by overproduction and cross-linking of fibrillar collagen (Types I and III) by activated cancer-associated fibroblasts (CAFs) (Najafi et al., 2019).

Available *ex vivo* and engineered-substrate data indicate that tumor-associated stroma may reach stiffness values of 15–50 kPa, nearly ten-fold greater than that of healthy lung tissue. Importantly, these readings are largely obtained from atomic force microscopy (AFM) measurements of resected tissue sections and polyacrylamide- or polydimethylsiloxane (PDMS)-based *in vitro* substrate assays. Accordingly, they cannot be uncritically extrapolated to *in vivo* tumor stiffness or local cellular strain as these *in vivo* mechanical properties are shaped by 3D nonlinear viscoelasticity, fractal tortuosity, and ECM stress relaxation (Guo et al., 2022; Deng et al., 2025). Findings from mechanobiology suggest that such pathological matrix stiffening can trigger multiple pro-oncogenic signaling cascades. High matrix rigidity functions as a physical ligand to drive integrin clustering on the plasma membrane and activate the Rho/ROCK signaling axis (Paszek et al., 2005). The downstream tension-dependent signaling facilitates focal-adhesion formation and stress-fiber assembly, which further sustains cell proliferation and survival through the Yes-associated protein/transcriptional co-activator with PDZ-binding motif (YAP/TAZ) transcriptional co-activators (Dupont et al., 2011; Ishihara and Haga, 2022). Aligned with recent mechanometabolic research, elevated substrate stiffness is associated with higher dynamin-related protein 1 (DRP1) expression, which may subsequently promote mitochondrial fragmentation (MacVicar and Langer, 2021; Ke et al., 2023). Stiffness-evoked mitochondrial dysfunction is, therefore, hypothesized to represent one plausible early link within the mechanochemical-coupling cascade, translating physical mechanical stimuli into intracellular oxidative stress.

#### 2.1.2 Pathological cyclic stretch: the high-magnitude strain threshold

In addition to the “static” resistance of matrix stiffness, pathological cyclic stretch represents a form of dynamic mechanical stimulation of lung cells. Physiological rhythmic stretching (typically under 5%–10% strain) ensures alveolar surfactant secretion and maintains epithelial homeostasis (Doryab et al., 2019). High-magnitude strain results from aberrant mechanical perturbation, compounded by disordered breathing or tumor-induced local solid stress, and leads to adverse cellular responses (Birukov et al., 2003).

A review of *in vitro* literature shows that a cyclic stretch of ∼20% has been widely employed as an experimental model of pathological mechanical stress. The mechanical deformation at this magnitude exceeds the adaptive capacity of alveolar epithelial cells (AECs) in two-dimensional elastomeric membrane assays (Chapman et al., 2005). Within this experimental paradigm, 20% cyclic stretch is capable of triggering a rapid and sustained oxidative burst. This response, as characterized in mechanobiological research, appears to represent a mechanochemical cascade that begins with mechanical perturbation of the cell membrane and activates the membrane-bound NOX2. However, it is critical to qualify that 20% cyclic stretch on flat 2D elastomeric (PDMS/silicone) membranes represents an extreme, hyper-physiological strain model closer to acute ventilator-induced lung injury (VILI) or barotrauma, rather than chronic low-magnitude strains occurring in solid tumors *in vivo* (Zitnay et al., 2022). *In vivo*, 3D nonlinear viscoelastic matrix strain, fractal tortuosity, and ECM stress relaxation buffer physical forces such that local cell strain in routine tumor progression likely remains substantially lower (Kraning-Rush et al., 2011). Thus, the 20% threshold should be interpreted as an experimental upper-bound strain model used to uncover mechanosensitive pathways, not as a universal, conserved cancer-associated threshold (Chapman et al., 2005; Tanaka et al., 2017).

In addition, this experimental strain paradigm is able to alter the delicate alveolar–capillary barrier *in vitro*. Mechanical dissociation of tight junction proteins like zonula occludens-1 (ZO-1) and claudins can occur under high-magnitude tension, causing an increase in paracellular permeability (Cavanaugh and Margulies, 2002; Jacob and Gaver, 2012). This disruption of the barrier may be exacerbated by the synergistic effects of physical strain and oxidative stress: ROS produced during high-magnitude strain directly affect the integrity of junctional complexes, further driving disruption of barrier integrity (Rao, 2008; Song et al., 2016). Such structural changes may allow inflammatory cells to migrate into the area and, as current mechanistic evidence suggests, may create a permissive environment for further metastatic migration.

There is growing evidence that oxidative damage caused by experimental strain is not fleeting. Prolonged incubation with 20% cyclic stretch *in vitro* results in accumulation of the canonical 8-hydroxy-2′-deoxyguanosine (8-OHdG) oxidative DNA damage marker and an increase in the secretion of the alarmin HMGB1 (Mayr et al., 2002; Kim et al., 2022). With secondary oxidative insults, HMGB1 expression may be substantially upregulated, as suggested by the existing mechanistic data. Thus, pathological cyclic stretch in experimental models may serve as a fundamental mechanistic factor in the mechanochemical cascade, supplying repetitive mechanical stimuli that can prime cells for conditions favoring chronic genomic instability (Gorini et al., 2021; Kim et al., 2024).

### 2.2 Mechanosensing at the nuclear envelope: the “last mile” of force transduction

Mechanical signals do not stop in the cytoplasm but may eventually converge on the nucleus, which acts as a central hub controlling genomic integrity. The discovery of the nucleus as a key mechanosensor has transformed the perception of how mechanical stress influences cell fates (Kirby and Lammerding, 2018). Within the lung TME, where high-magnitude experimental strain and elevated matrix stiffness are applied to cells in engineered models, the nuclear envelope may represent the “last mile” of force transduction, potentially converting extranuclear mechanical tension into chromatin remodeling and downstream transcriptional reprogramming (Morival et al., 2025).

#### 2.2.1 The LINC complex and nuclear rigidity

The Linker of Nucleoskeleton and Cytoskeleton (LINC) complex forms physical connections between the ECM and the nucleoskeleton and consists of SUN-domain proteins and KASH-domain Nesprins (Sosa et al., 2013; Tapley and Starr, 2013). This continuous mechanical bridge allows mechanical signals that are perceived by integrins to travel rapidly along the actin cytoskeleton to the nuclear periphery, much faster compared to diffusion of chemical signals (Lombardi et al., 2011; Hao and Starr, 2019). In cancer cells residing within stiffer stromal microenvironments, mechanical tension—a function of LINC assembly—flattens nuclei and pre-stresses the nuclear envelope (Lovett et al., 2013; Wang T.-C. et al., 2024). Based on the current mechanistic understanding, this mechanical remodeling makes the nucleus more vulnerable to structural damage under sustained biomechanical loading conditions.

#### 2.2.2 Lamin A/C: the molecular strain gauge

Lamin A/C, a type V intermediate filament protein, is located at the center of the nuclear mechanoadaptation process, building a meshwork in the inner nuclear membrane. Lamin A/C acts as a biological “strain gauge”: it undergoes tight assembly and subsequent phosphorylation in response to external mechanical stress with high sensitivity (Osmanagic-Myers et al., 2015; Donnaloja et al., 2020).

Under experimental cyclic stretch (similar to 20% magnitude in 2D models), considerable tensile strain in the nuclear envelope may occur when lung epithelial cells are stretched. In this state, Lamin A/C molecules may undergo conformational changes in response to force, exposing specific “cryptic” sites and potentially altering the binding affinity of lamins for various transcription factors (TFs) and chromatin-modifying enzymes. High tension has been demonstrated to cause Lamin A/C dephosphorylation and result in the formation of a more rigid lamina that protects the genome from mechanical rupture (Buxboim et al., 2014; Stephens et al., 2017). However, under chronic tumor-associated stress, this reparative pathway may malfunction—leading to paradoxical outcomes, as excessive stiffening of the nucleus could potentially promote genomic instability by reducing the mobility of DNA repair foci (Bell and Lammerding, 2016; Bosch-Calvet et al., 2025). It remains a theoretical paradigm awaiting direct *in vivo* validation whether these nuclear mechanical changes directly cause fixed driver mutations in human lung cancer. These multi‑layered mechanical signal‑transmission events are summarized in Figure 1.

**FIGURE 1:**
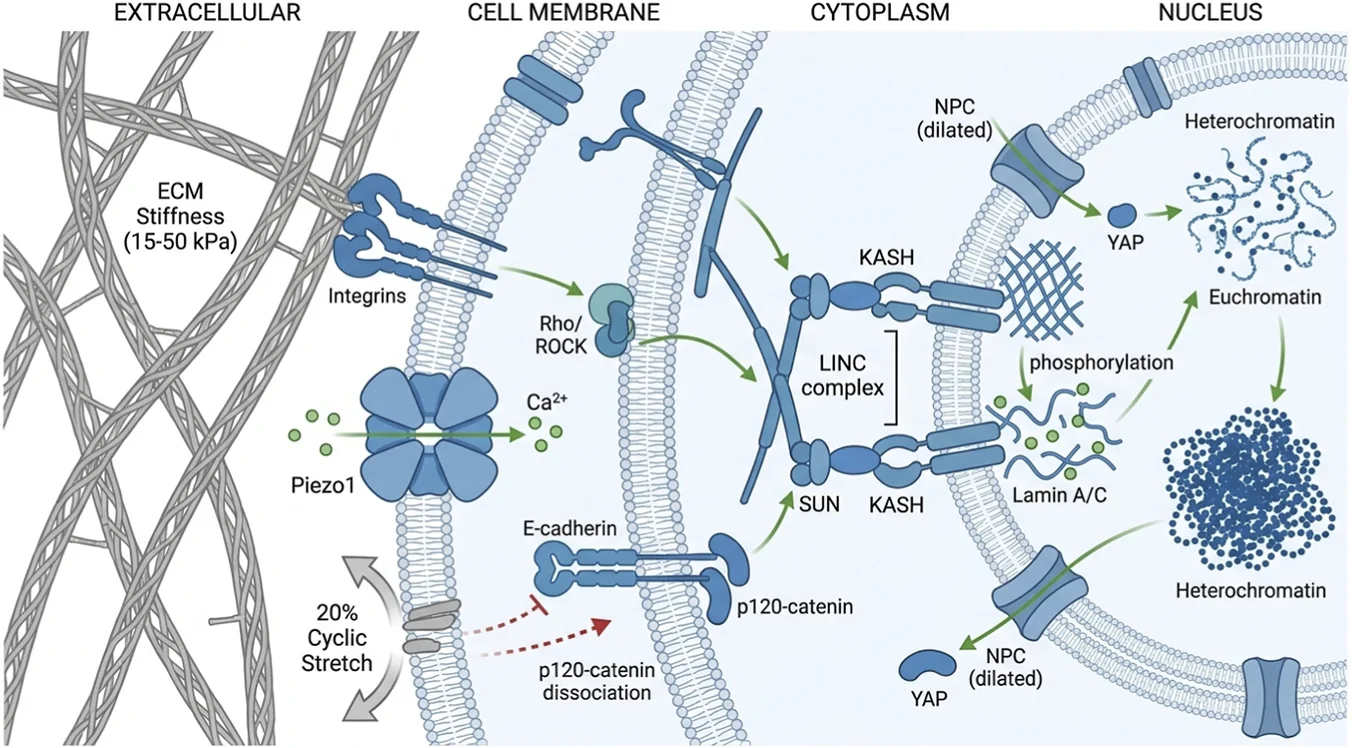
Biomechanical landscape and nuclear mechanotransduction.

#### 2.2.3 Regulation of nuclear pore complex and nucleocytoplasmic transport

Nuclear pore complexes (NPCs) are directly modulated by the mechanical properties of the nuclear lamina. Pathological tensile force on the nuclear envelope may expand the diameter of the NPC’s central channel, stretching the NPC scaffold (Elosegui-Artola et al., 2017). Mechanical dilation of NPCs may increase passive molecular permeability, which could allow large proteins to enter the nucleus, such as mechanosensitive TF YAP, to a degree that is independent of the requirement for canonical nuclear localization signal (NLS) (Zimmerli et al., 2021; Andreu et al., 2022).

In lung adenocarcinoma cells studied *in vitro*, mechanochemical coupling has been observed between alterations in mechanical tension and the transcription program associated with cell cycle progression and EMT (Donnaloja et al., 2019; Park et al., 2024). Based on the current mechanobiological consensus, this bypass of traditional nucleocytoplasmic transport checkpoints may represent an important cellular vulnerability that compromises the maintenance of a stable transcriptome in the face of chronic mechanical loading.

#### 2.2.4 Chromatin state and genomic fragility

The most striking effect of nuclear mechanosensing may be chromatin remodeling itself. Lamin A/C associates with lamina-associated domains (LADs) that are usually transcriptionally silent heterochromatin (van Steensel and Belmont, 2017). The mechanical stress induced during experimental pathological stretch may be transmitted directly to these heterochromatin regions, which may switch from a condensed to a more relaxed euchromatin state (Nava et al., 2020; Jabre et al., 2025).

This chromatin relaxation could be involved in a rapid chromatin transcriptional response to mechanical stress, but available mechanistic evidence indicates it may primarily protect against mechanical damage, rather than inflict damage—a distinction that becomes critical in the context of cancer (Srinivas et al., 2019; Anlaş and Nelson, 2020; Dos Santos et al., 2021). However, in situations of chronic stress, such decondensation might render certain parts of the genome vulnerable to ROS. High oxidative stress combined with relaxation of the physical chromatin structure could generate conditions that favor DNA DSBs and chromosomal rearrangements. Thus, Lamin A/C functions not only in force sensing but also in protecting genomic stability. The mechanical dysfunction of Lamin A/C resulting from pathological stretch may play an important role in genomic instability, a hallmark of lung cancers during evolution, though direct causal evidence in intact tissue remains to be established (Donnaloja et al., 2020; Bellanger et al., 2022).

This conceptual schematic illustrates how pathological mechanical forces in the lung TME may be transmitted to the nucleus based on *in vitro* and *ex vivo* evidence. The left panel shows two mechanical inputs: matrix stiffening (15–50 kPa, measured by AFM in excised tissues and engineered substrates) and ∼20% cyclic stretch (experimental 2D elastomeric membrane model). These forces may activate membrane-bound mechanosensors—Piezo1 (triggering Ca^2+^ influx) and integrins (activating Rho/ROCK)—while simultaneously disrupting adherens junctions through p120–catenin dissociation from E-cadherin. The LINC complex may relay these signals to the nuclear envelope, where Lamin A/C phosphorylation may induce NPC dilation and chromatin remodeling from condensed heterochromatin to relaxed euchromatin, potentially facilitating YAP nuclear entry. Green arrows indicate activation; red dashed arrows indicate disruption; blue indicates structural proteins; gray indicates mechanical forces. This figure represents a conceptual pathway synthesis rather than plotted experimental datasets.

## 3 The oxidative engine: a proposed model for translating mechanics into chemistry

The conversion of physical forces into pathological biochemical signals constitutes a proposed hallmark of lung cancer initiation. This “mechanochemical transduction” is hypothesized to manifest primarily as rapid, localized ROS production (Chatterjee and Fisher, 2014; Hou et al., 2024). As characterized in experimental systems, this oxidative burst is not an unspecific stress response; instead, it represents a tightly regulated reaction with a defined mechanical threshold and dedicated enzymatic machinery, as demonstrated in lung mechanobiology studies (Browning et al., 2012).

### 3.1 The mechanical threshold: NOX2 activation as a biochemical switch (*in vitro* evidence)

The central component of the proposed mechanochemical engine is NOX2, a multi-subunit enzymatic complex traditionally known for its role in the respiratory burst of phagocytes, but which is now emerging as an important mechanical transducer in the lung epithelium in experimental settings (Browning et al., 2012). The activation of NOX2 as a biochemical switch is conceptually important and is triggered mainly when mechanosensation exceeds homeostatic physiological limits (Chapman et al., 2005; Brandes et al., 2014). It is essential to clarify that studies within this field have measured the activities of multiple forms of NADPH oxidases, including total NADPH oxidase, NOX1, NOX2, and NOX4. The specific isoform mediating the observed effects may vary according to the cell type and stimulus. The following discussion focuses primarily on NOX2 as this isoform has been most frequently implicated in high-magnitude stretch responses in AECs, while acknowledging that other isoforms may contribute in distinct contexts.

#### 3.1.1 The ∼20% strain paradigm: an experimental upper-bound model

The respiratory cycle of the lung involves cyclic stretching, and strain magnitude is a critical determinant of the subsequent cell fate. Published *in vitro* study results validated a conserved bimodal cellular response to mechanical strain (Birukov et al., 2003; Dick et al., 2013). Physiological homeostasis is maintained for low-magnitude stretch (5%–10%), with strain magnitudes at or above 15% initiating pathological responses in 2D culture systems, and the 20% cyclic stretch commonly representing the pathological range in mechanobiological studies (Chapman et al., 2005).

When the plasma membrane is subjected to 20% cyclic stretch on elastomeric substrates, its deformation may be sufficient to initiate rapid assembly of the NOX2 enzymatic complex. This process involves recruitment of cytosolic components (p47phox, p67phox, and Rac1) to membrane-bound cytochrome b558, comprising gp91phox and p22phox (Kou et al., 2009; Liang et al., 2019). Mechanical stretching of the phospholipid bilayer, which may cause spatial conformational changes in these subunits, could reduce the energy barrier for complex assembly. Consistent with a rapid, non-transcriptional mechanochemical cascade, superoxide (O_2_•^-^) production bursts occur within minutes of 20% stretch, before detectable changes in transcription, consistent with a direct mechanical-to-enzymatic conversion process *in vitro* (Zhao et al., 2024). We emphasize that this threshold reflects the experimental conditions of 2D elastomeric membranes and should not be presented as a universal *in vivo* cancer threshold.

#### 3.1.2 Mechanosensitive signaling cascades leading to NOX2 assembly

This stretch-induced activation of NOX2 in experimental models is not an isolated phenomenon but instead is regulated by the action of upstream mechanosensors. In particular, high-magnitude tension activates the phosphoinositide 3-kinase (PI3K) pathway and its downstream target Akt, which have been established as key regulators of NOX2 activation, with evidence indicating that they regulate phosphorylation of p47phox, an essential step for assembly of NOX2 (Hoyal et al., 2003; Sedding et al., 2005). This phosphorylation causes a conformational change in p47phox, which enables it to interact with p22phox and enable complex formation.

Furthermore, mechanical force exerted on integrins, the main linkages between the cell and stiffened ECM, triggers activation of a family of small GTPases, in particular Rac1 (Akhiani and Martner, 2022; Jiang Y. et al., 2022). The mechanical activation of Rac1 may be essential to the activation of the NOX2 complex and promotes translocation of cytosolic subunits to the membrane. Existing mechanobiological studies are consistent with the idea that “pre-stress” on integrins reduces the threshold for Rac1 activation (Lee et al., 2012), thereby potentially rendering cells hypersensitive to high-magnitude cyclic stretch applied during abnormal breathing or local tumor growth in experimental settings.

#### 3.1.3 Localized oxidative microdomains and “ROS-Induced ROS release”

One important aspect of NOX2-mediated mechanotransduction, as characterized *in vitro*, is that it generates localized oxidative microdomains. Whereas exogenous oxidative stress is distributed throughout the cell, ROS generated by mechanochemistry in experimental systems may be localized to the plasma membrane and sub-membranous actin cytoskeleton, consistent with NOX2 localization (Brandes et al., 2014; Nordzieke and Medraño-Fernandez, 2018).

This localized production of ROS may set up a “feedback loop” called ROS-induced ROS release (RIRR). The primary superoxide produced by NOX2 is dismutated into hydrogen peroxide (H_2_O_2_), which diffuses to neighboring organelles, including the mitochondria. This cross-talk is essential; existing evidence has shown that NOX-induced ROS may open mitochondrial permeability transition pores (mPTP), resulting in release of mitochondrial ROS (mtROS) in a positive feedback loop (Zorov et al., 2006; Kim Y.-M. et al., 2017). It is hypothesized that this amplification loop converts a localized physical stimulus to a systemic intracellular oxidative response capable of driving the long-term chemical energy necessary to affect genomic integrity.

#### 3.1.4 Impacts on the antioxidant defense system: the Nrf2 conflict and temporal dynamics

When ROS levels reach the high-magnitude stretch threshold in experimental models, an oxidative burst occurs, causing profound redox imbalance. The Nrf2-ARE (Antioxidant Response Element) pathway represents a physiological stress response mechanism that maintains redox balance under the conditions of physiological strain. Nrf2 is a master transcription factor that activates antioxidant enzymes such as superoxide dismutase (SOD) and glutathione peroxidase (GPx), which are well recognized to protect tissues subjected to mechanical stress (Papaiahgari et al., 2007; Fragoulis et al., 2023).

However, growing mechanobiological research data suggest there exists a functional “tipping point” at which the mechanochemical oxidative burden overwhelms the Nrf2 system. It is hypothesized that excessive production of ROS due to NOX2 may alter Nrf2-mediated antioxidant defense by interfering with its synthesis, nuclear transport, or activity (Papaiahgari et al., 2007; Li et al., 2009). This functional inhibition of the adaptive antioxidant response may effectively eliminate the “antioxidant brake” and allow for unrestrained operation of the mechanochemical engine, causing cumulative damage to proteins, lipids, and ultimately DNA (Juan et al., 2021).

It is critical to reconcile the Nrf2 paradox: oxidative stress classically activates Nrf2 as an adaptive survival response. The precise mechanical, temporal, and spatial conditions under which pathological tension suppresses Akt/Nrf2 repair pathways versus when it induces them remain incompletely defined. Acute mechanical stress may transiently activate Nrf2 as a protective response, whereas chronic, high-magnitude cyclic stretch may exhaust this adaptive capacity, leading to paradoxical suppression. The transition from Nrf2 activation to Nrf2 dysfunction may depend on the duration and amplitude of mechanical stress, the specific cell type, and the concurrent inflammatory milieu—factors that require further systematic investigation.

#### 3.1.5 Quantifying the oxidative load: clinical and experimental evidence

This oxidative burst can be quantitatively characterized in experimental models. *In vitro* models validate that 20% cyclic stretch induces a significant increase in dihydroethidium (DHE) staining, a marker for superoxide accumulation, and an increase in 4-hydroxynonenal (4-HNE), a marker of extensive lipid peroxidation (Chapman et al., 2005). These molecular readouts may be considered signatures of mechanical force transduction in experimental systems because they are validated oxidative stress markers (Barrera, 2012; Zhong and Yin, 2015). In clinical scenarios, areas of increased mechanical tension in lung cancer tissues have been associated with increased expression of oxidative markers, especially at the tumor’s invasive front (Zemskov et al., 2019). This congruence provides conceptual validation of the potential clinical relevance of NOX2-mediated mechanochemical signaling pathways, though direct causal links in human disease remain to be established.

### 3.2 The molecular switch: p120-catenin, macromolecular crowding, and mitochondrial homeostasis

The initial oxidative burst is driven by NOX2 in experimental models, but sustained operation of the proposed mechanochemical engine may require deep metabolic rewiring and destabilization of organelles. In lung epithelial cells, the p120-catenin complex may act as a high-fidelity molecular switch that controls this regulatory transition (Liu et al., 2019). p120-catenin is not only a structural adhesion stabilizer but also serves as a “rheostat” that may regulate both mechanical integrity of the tissue and mitochondrial redox homeostasis (Wang Y. et al., 2011). Pathological high-magnitude tension may cause disruption and depletion of p120-catenin, potentially contributing to the transition of epithelial cells from a state of transient mechanical stress to a persistent pro-oncogenic oxidative microenvironment (Inumaru et al., 2009; Gu et al., 2016).

To enhance biophysical rigor, it is important to consider that physical strain and matrix stiffening alter cell volume, cytosolic hydration, and macromolecular packing, which directly govern the spatial condensation and assembly of signaling hubs. Recent advances in biophysics indicate that macromolecular crowding and biomolecular phase separation (condensate formation) are critical determinants of signaling pathway activation (Alberti and Dormann, 2019; Xie et al., 2026). Under pathological mechanical stress, changes in cytoplasmic osmotic pressure and cytoskeletal tension may alter the phase behavior of multi-protein assemblies such as the p120-catenin/Toll-like receptor 4 (TLR4) hub, DRP1 mitochondrial fission complexes, and NLRP3 inflammasomes. These structures may not assemble strictly through classical linear “lock-and-key” phosphorylation cascades alone but may also form through liquid–liquid phase separation (LLPS) driven by changes in the local protein concentration, molecular crowding, and intracellular hydration. Mechanical compression or stretch may, thus, regulate signaling not only by activating kinases but also by physically altering the condensate-forming properties of key adapter proteins, a dimension that warrants incorporation into future mechanochemical models. This phase behavior could serve as a fast, non-enzymatic “switch” for signal integration, operating in parallel with classical phosphorylation cascades. It should be noted, however, that the potential role of LLPS in lung cancer mechanotransduction is largely understudied, and direct evidence in native lung tumor tissue is currently unavailable (Sun et al., 2024); this represents an important area for future investigation.

#### 3.2.1 The mechanical failure of the adherens junction and p120-catenin dissociation

In healthy alveolar epithelium, p120-catenin is sequestered at the plasma membrane where it associates with the juxtamembrane domain of E-cadherin and blocks its internalization and degradation (Davis et al., 2003). This sequestration is necessary to maintain the “mechanical silence” of the cell, thereby preserving epithelial barrier integrity by stabilizing adherens junctions. However, the mechanical force exerted on adherens junctions is exponentially greater under pathological cyclic stretch or under the constant “pull” of a stiffened ECM (15–50 kPa in engineered substrates).

Results from mechanically induced lung injury studies suggest that an overactive tension causes the dissociation of p120-catenin from E-cadherin, likely through phosphorylation of p120-catenin (Wang Y. et al., 2011). In both experimental contexts, this mechanical dissociation may result in the release of p120-catenin into the cytoplasm, where it is rapidly ubiquitinated and degraded or translocated to the nucleus, findings consistent with observations of p120-catenin turnover and E-cadherin endocytosis under cyclic stretch conditions (Gu et al., 2016). Loss of the junctional pool of p120-catenin may represent a key “sensor” of mechanical failure. The epithelial barrier integrity may be lost in the absence of p120-catenin, and crucially, the cell’s main inhibitory brake on multiple pro-oxidative signaling cascades may be lost (Liu et al., 2019).

#### 3.2.2 The p120-catenin/TLR4 axis: a mechanochemical gateway

The most important consequence of p120-catenin loss is unchecked activation of the TLR4 signaling pathway. Under physiological conditions, p120-catenin negatively regulates TLR4 by preventing its assembly into active signaling complexes (Yang et al., 2014). When p120-catenin is mechanically depleted in experimental models, this inhibition may be removed, allowing MyD88 recruitment and activation of nuclear factor kappa-B (NF-κB) and mitogen-activated protein kinase (MAPK) pathways (Wang Y.-L. et al., 2011).

This p120–TLR4 axis represents a proposed example of mechanochemical coupling: a physical event (disruption of junctions) may initiate a biochemical cascade that activates NADPH oxidase subunits and induces proinflammatory cytokine activity (Liu et al., 2015). In the context of lung cancer evolution, this pathway may establish an “oxidative” environment, consistent with observations that ROS signaling through TLR4 can promote lung tumor progression (Velasco et al., 2023). The production of ROS by TLR4 may not be restricted to the membrane but may be distributed throughout the cytosol, thus establishing a second line of attack on the mitochondria, which is observed in TLR4-activated cancer models (Yuan et al., 2013). However, it must be emphasized that this axis has been demonstrated primarily in VILI and inflammatory models; its direct validation as a continuous causal sequence in spontaneous lung carcinogenesis remains an open empirical question.

#### 3.2.3 DRP1-mediated mitochondrial fragmentation: the signal amplifier

Increasing ROS levels in the cell may trigger a dramatic change in the morphology and function of the mitochondria. They are highly dynamic, existing in equilibrium between fusion (elongation) and fission (fragmentation). Loss of p120-catenin may tip the balance toward excessive fission, as may pathological mechanical stress (Liu et al., 2019).

DRP1 is the orchestrator of this process. In response to mechanochemical stress in experimental systems, DRP1 is phosphorylated at particular serine residues, moving from the cytosol to the mitochondrial outer membrane (Wang Y. et al., 2020). DRP1 forms spirals here that physically constrict the mitochondria. This fission appears to be directly triggered by ROS produced via the p120–TLR4 axis, as TLR4 activation has been shown to lead to DRP1-dependent mitochondrial fragmentation (Kapetanovic et al., 2020). The fragmented mitochondria are not merely diminutive; they are functionally impaired. Their loss of membrane potential leads to excessive electron “leakage” from the electron transport chain (ETC.), mainly through Complexes I and III (Ahmed et al., 2019). This leakage leads to excessive increase in mitochondrial ROS (mtROS), and evidence indicates that DRP1-dependent fission may be necessary for oxidative signaling and ROS production (Röth et al., 2014).

#### 3.2.4 mtROS as a mechanochemical signal multiplier

The mtROS generated by DRP1-mediated fragmentation may act as effective signal multipliers. In contrast to NOX2-derived ROS, which are short-lived and localized to specific areas, mtROS may have broader regulatory effects within the cell because they are close to core metabolic and genetic machinery (Dan Dunn et al., 2015; Romani et al., 2022). A major consequence of mtROS accumulation may be potent activation of the NLRP3 inflammasome by mtROS and leaked oxidized mtDNA, leading to release of proinflammatory alarmins such as HMGB1 (Shimada et al., 2012).

All compiled findings support a possible synergy loop: mechanical stress may lead to p120 loss, which activates TLR4, which stimulates ROS formation, which causes DRP1 fission, which stimulates further activation of TLR4 and NOX2, which eventually stimulates more p120 loss (Wu et al., 2013). This self-reinforcing vicious cycle is a plausible explanation for why even brief exposure to 20% stretch *in vitro* may leave an “oxidative scar” on the cell, pushing it toward conditions favoring persistent genomic instability. Nevertheless, this loop remains a theoretical model requiring validation in intact three-dimensional tissue systems. This positive‑feedback oxidative cascade is illustrated in Figure 2.

**FIGURE 2:**
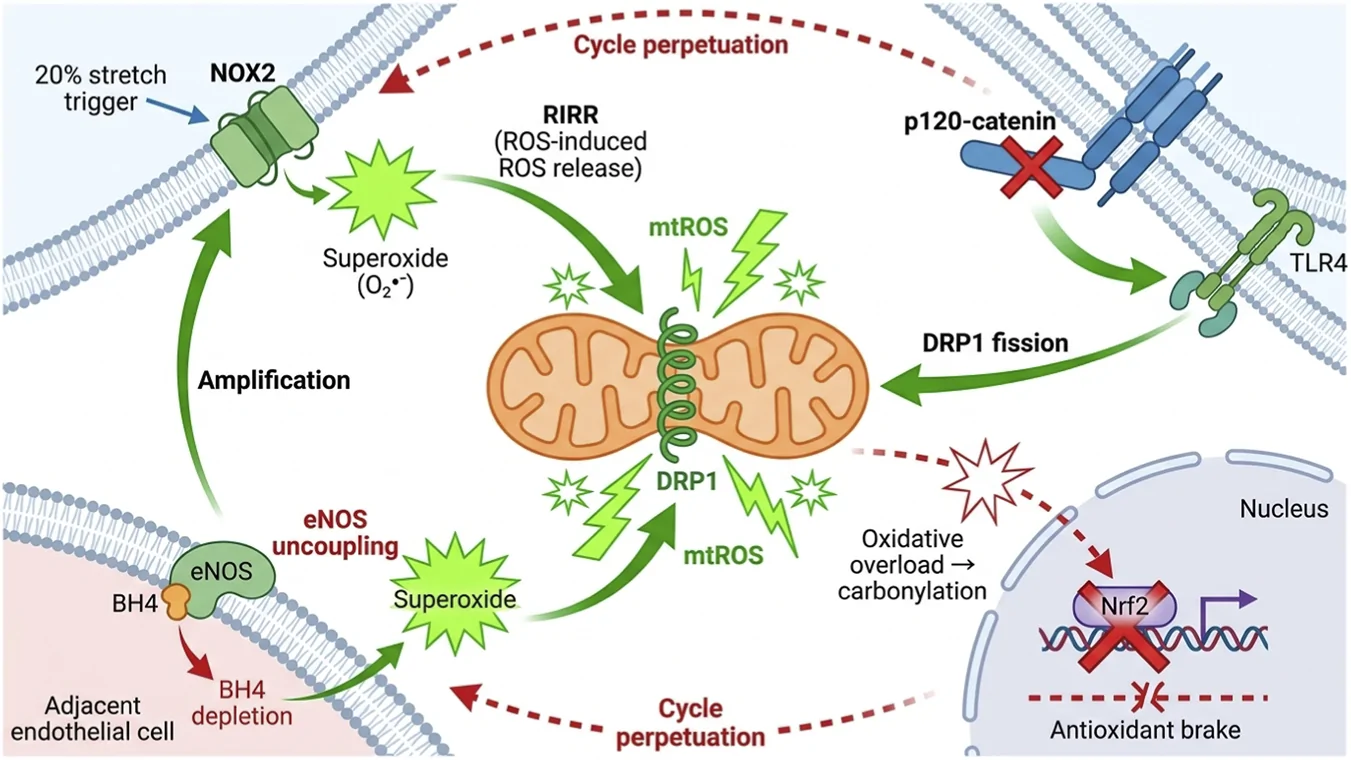
The oxidative engine and mitochondrial amplification loop.

#### 3.2.5 Implications for malignant evolution: from homeostasis to collapse

Finally, the p120-catenin/DRP1 axis may represent a molecular point of no return in tumor progression. In healthy tissue, p120-catenin functions to sense and transmit mechanical signals properly so as to maintain tissue homeostasis (Yu et al., 2011; Kourtidis et al., 2013). Mechanical disruption of junctional integrity and p120-catenin dysfunction may effectively unleash mitochondrial dynamics that allow the mitochondria to cause genome-damaging oxidative stress in malignant cells in the microenvironment of lung cancers (Yang et al., 2018).

The “Oxidative Engine” is proposed not merely as a collection of metabolic enzymes but as a complex organelle-regulated system that is modulated by tissue physical properties. DRP1-regulated mitochondrial fragmentation may maintain high levels of mtROS, contributing to sustained oxidative stress. This stress may drive DNA damage, chromosomal instability, and eventual malignant transformation, hallmarks of aggressive lung cancer (Rehman et al., 2012; Sabharwal and Schumacker, 2014).

### 3.3 eNOS uncoupling: a cross-disciplinary extrapolation from vascular mechanics to lung pathology

The vascular endothelium and epithelial cells of the lung are not only barriers but also active players in the mechanochemical signaling network within this complex microenvironment. Endothelial Nitric Oxide Synthase (eNOS) is a key player in this network. In healthy tissue, eNOS releases nitric oxide (NO), which is considered a potent vasodilator and anti-inflammatory signal molecule, maintaining vascular and alveolar homeostasis (Siragusa and Fleming, 2016; Janaszak-Jasiecka et al., 2023).

While eNOS uncoupling is well-documented in vascular endothelial mechanobiology, cardiovascular research, and VILI models, direct evidence supporting its role as a primary driver of parenchymal lung epithelial genomic instability in human lung cancer is still lacking. The discussion below draws on established vascular endothelial mechanisms to build a hypothesis-generating framework for the lung cancer microenvironment. Notably, cell-type-specific differences in eNOS expression, tetrahydrobiopterin (BH4) metabolism, and mechanotransduction may considerably modify the functional relevance of this pathway across distinct pulmonary cell populations.

#### 3.3.1 The structural basis of eNOS: coupling and uncoupling

The molecular architecture of eNOS is highly delicate. The active eNOS is a homodimer, which is crucial for transferring electrons from the reductase domain of one monomer to the oxygenase domain of the other (Andrew and Mayer, 1999; Gielis et al., 2011). Electron flow requires the cofactor tetrahydrobiopterin (BH4) and the substrate L-arginine. For the intact dimer, in the presence of BH4, the terminal oxygen is reduced to form NO (Landmesser et al., 2003; Berka et al., 2004).

Pathological mechanical tension may disrupt this well-organized structure. This high-magnitude physical strain may alter the conformational state of eNOS or disrupt the dimeric microenvironment, thereby uncoupling electron transfer from NO synthesis. While this process has been suggested to involve eNOS monomerization, recent evidence indicates that heme domain dysfunction and BH4 depletion, rather than monomerization, are the primary drivers of uncoupling (Gebhart et al., 2019). In this uncoupled state, electrons that would otherwise reduce L-arginine are instead used to produce superoxide (O_2_•^-^) rapidly and continuously. This structural change leads to conversion of a vasoprotective enzyme into a potent superoxide generator, thus potentially supplying chemical “fuel” for the mechanochemical engine (Bendall et al., 2005; Luo et al., 2014).

#### 3.3.2 Mechanotransduction pathways triggering eNOS dysfunction

Specific mechanosensors in the lung may relay physical signals to eNOS, mediating the lung’s response to abnormal tension (Migulina et al., 2023). With high-magnitude stretch, plasma membrane tension may be sensed by mechanosensitive ion channels and integrin-linked kinases that directly regulate eNOS activity through post-translational modifications (Janaszak-Jasiecka et al., 2023).

One of the main mechanisms is the mechanical activation of Protein Kinase C (PKC) and NADPH oxidases (NOXs). As described in Section 3.1, ROS released from NOX2 in response to high-magnitude stretch represent the initial oxidative pulse in experimental models (Wu et al., 2014; Jiang et al., 2025). These NOX-derived superoxide radicals rapidly react with pre-existing NO to create a highly reactive nitrogen species called peroxynitrite (ONOO^−^). Peroxynitrite is considered one of the most important mediators capable of disrupting eNOS coupling by oxidizing the essential cofactor BH4 to BH2 (Förstermann and Münzel, 2006; Gielis et al., 2011). The end effect of mechanical stress may, therefore, be BH4 depletion, which is thought to be the final biochemical event that “silences” eNOS, locking it in an uncoupled, ROS-producing state and, thus, potentially generating a persistent source of oxidative stress even after mechanical stress has subsided.

#### 3.3.3 Synergistic impact of matrix stiffness and solid stress

Along with the dynamic trigger of cyclic stretch, the lung stroma in tumor regions may also be chronically preloaded, often with a stiffness of 15–50 kPa in experimental measurements, which may sensitize eNOS to uncoupling (Xu et al., 2023; Mierke, 2024). A stiffened ECM promotes strong focal adhesion assembly and stress fiber formation, while eNOS may become anchored to the cytoskeletal network via integrin-mediated mechanotransduction (Paszek et al., 2005; Jannatbabaei et al., 2020).

This cytoskeletal tethering may impair the mobility of eNOS and regulate its functional interaction with other regulatory partners, including caveolin-1. In the presence of increased mechanical tension, prolonged contact with inhibitory scaffold proteins such as caveolin-1 may further impair NO bioavailability and promote uncoupled electron leakage that enhances superoxide generation (Lobysheva et al., 2011; Łuczak et al., 2020). Together, the static mechanical constraint from matrix stiffening and the dynamic insult of pathological cyclic stretch may act in concert to trigger endothelial dysfunction, potentially creating a highly localized oxidative microenvironment at the tumor–vessel interface (Kohn et al., 2015; Wu and Birukov, 2019).

#### 3.3.4 From eNOS uncoupling to genomic instability: a hypothesized feedback loop

Uncoupled eNOS produces ROS and peroxynitrite, which may exert significant biological effects on neighboring pulmonary cells (Gielis et al., 2011). Unlike the relatively localized ROS produced by NOX2, the products of eNOS uncoupling may be more chemically stable and able to cross cell membranes to affect neighboring epithelial cells and even enter the nuclear compartment (Szabó and Ohshima, 1997; Islam et al., 2015).

This transmembrane diffusion may trigger a secondary cascade of oxidative DNA damage. Peroxynitrite has been extensively reported to induce DNA base modifications and strand breaks and may influence the activity of DNA repair enzymes under pathological redox conditions through oxidative nitration (Szabó, 1996). Furthermore, bioavailable NO, an intrinsic inhibitor of several proinflammatory signal transduction pathways, may be depleted, potentially permitting unrestricted NF-κB activation and elevated HMGB1 expression under oxidative and mechanical stress conditions (Wang J. et al., 2020; Zhang et al., 2023). This entire cascade represents a hypothesized self-propagating cycle: mechanical stress may promote eNOS uncoupling, leading to oxidative DNA damage and inflammatory activation. Chronic inflammation, in turn, may drive tissue remodeling through fibrosis and edema, which increases matrix stiffness and thereby re-stresses the tissue, amplifying the initial mechanical insult (Szabó and Ohshima, 1997; Liu et al., 2010; Freeberg et al., 2021).

#### 3.3.5 Therapeutic implications: re-coupling the engine

Use of mechanopharmacological to target eNOS uncoupling as a mechanochemical event opens new avenues for research (Linke et al., 2024; Gargalionis et al., 2025). Several proposed strategies supported by current evidence could theoretically reverse this dysfunctional state. L-arginine supplementation or BH4 precursors have been shown to improve eNOS coupling efficiency and restore NO production in various experimental conditions (Bevers et al., 2006; Förstermann and Münzel, 2006; Li and Forstermann, 2014). Furthermore, Nrf2 activators such as resveratrol have been shown to stabilize cellular antioxidant defenses, and quercetin is expected to have similar protective effects, which may prevent oxidative BH4 depletion and maintain the structural and functional integrity of eNOS (Ungvari et al., 2010; Xia et al., 2010; Zhang L. et al., 2024).

The critical threshold for pathological mechanical tension to induce eNOS uncoupling is of potential translational importance for lung cancer patients, especially those subjected to excessive mechanical stress due to tumor growth or clinical intervention (Kalli et al., 2023; Zhang L. et al., 2024). Targeting the eNOS-mediated mechanochemical signaling pathway may represent a feasible conceptual approach to interrupt the pathological cascade from mechanical strain to malignant progression, offering potential mechanotherapeutic opportunities to modulate the tumor mechanical microenvironment, pending rigorous preclinical validation.

This conceptual schematic depicts proposed core mechanochemical signaling cascades organized around a positive feedback loop. At the plasma membrane, experimental ∼20% stretch may trigger NOX2 assembly and superoxide production. This initial oxidative burst may drive p120-catenin dissociation from adherens junctions, potentially releasing TLR4 signaling. Both NOX2-derived ROS and TLR4 may converge on the mitochondria, promoting DRP1-mediated fission and excessive mtROS release, which may feed back to further activate NOX2 (RIRR). Simultaneously, oxidative overload may silence Nrf2-mediated antioxidant defense, while eNOS uncoupling—extrapolated from vascular endothelial studies and driven by BH4 depletion—may convert this enzyme into an additional source of superoxides. Green indicates oxidative/activation; red indicates inhibition/damage; orange indicates mitochondria. This figure represents a conceptual pathway synthesis rather than plotted experimental datasets.

## 4 Genomic instability: from mechanochemical stress to conditions favoring mutation

The ultimate expression of the proposed mechanochemical engine may be the physical and chemical breakdown of genome integrity. As in other mechanobiology examples where mechanical forces lead to genomic disruption, DNA instability in lung cancer may not be a random phenomenon but rather a likely direct result of chronic mechanical–oxidative assault on DNA (Simi et al., 2015; Shah et al., 2021; Mammel and Hatch, 2022). However, it is crucial to differentiate between well-documented transient genomic changes (such as γH2AX foci and 8-OHdG) and the permanent genomic changes that give rise to permanent cancer driver mutations. Although mechanochemical stress is clearly a mutagenic environment that might help eventual fixation of mutations, the field still lacks direct evidence for a link between a threshold of mechanical stress and the appearance of canonical driver mutations (e.g., KRASG12C, EGFR exon 19 deletions, and TP53 missense mutations) in native lung tissue (Frankell et al., 2023). Future studies that utilize spatial multi-omics and deep-sequencing techniques of intact 3D matrices are warranted to fill this gap. This section examines how physical tension and chemical bursts act in synergy to directly dismantle the genetic blueprint, representing key mechanistic steps in cancer mechanobiology.

### 4.1 Physical stress-mediated genetic destruction: molecular mechanisms

The genomic landscape is protected by multiple layers of defense, from the nuclear envelope to the densification of chromatin (Nava et al., 2020; Dos Santos et al., 2021). However, pathological mechanical stress, under conditions analogous to the high-magnitude cyclic stretch established in cancer models, specifically targets these defenses, inducing “genomic fragility” (Kaushal and Freudenreich, 2019; Nava et al., 2020; Shim et al., 2026).

#### 4.1.1 Mechanical tension and nucleosome disassembly

The first line of genetic defense is the organization of DNA into nucleosomes. In physiological conditions, wrapping of DNA around histone octamers provides both structural stability and protection from oxidative attack. In contrast, mechanical force applied in pathological stretch in experimental models, at high magnitudes and cycles, may propagate directly to chromatin fibers from the Lamin A/C network (Nava et al., 2020; Bastianello et al., 2023; Jabre et al., 2025).

Forced Chromatin Relaxation: When high-magnitude tension is applied *in vitro*, heterochromatin (tightly packed) may transition to euchromatin (relaxed). The “mechanical unzipping” of the genome may not be random; preferential unzipping may occur at LADs, consistent with work demonstrating that mechanical forces can alter the organization of LADs in lung cells (Scott et al., 2023; Shiraishi et al., 2023).

Nucleosome Displacement: Biophysical evidence indicates that extreme mechanical strain can physically displace histones from the DNA backbone in single-molecule assays (Ngo et al., 2015; Díaz-Celis et al., 2022). This nucleosome “breathing” or disassembly may expose the naked phosphodiester backbone to high concentrations of ROS, which may occur because of activated NOX2 and mitochondria in the vicinity, and the documented relationship between mechanical stress and ROS production and DNA damage (Shah et al., 2021; Andrs et al., 2023).

Replication Fork Targeting: This mechanical tension may disrupt the activity of replication forks during the S-phase of the cell cycle. It is thought that this physical stress on the DNA template, coupled with chemical insult from ROS, may give rise to “replication stress” involving stalled and collapsed replication forks, which are known to be important triggers for chromosomal translocations (Berti et al., 2020; Wilhelm et al., 2020; Shah et al., 2021).

#### 4.1.2 The ROS-Induced DNA double-strand break cascade

High-magnitude cyclic stretch in experimental models (similar to strains that cause DNA damage in AECs) may cause chromatin relaxation, rendering DNA a high-affinity target for hydroxyl radicals (•OH) generated by the Fenton reaction (Upadhyay et al., 2003; Chapman et al., 2005; Zhao, 2023).

The Chemistry of the Break: ROS may attack the sugar–phosphate backbone, causing base loss (apurinic/apyrimidinic sites) and single-strand breaks (SSBs). These SSBs may rapidly convert into DSBs when they occur in close proximity or are generated during mechanical tension. Clustered oxidative lesions can lead to DSBs (Gorini et al., 2021; Hahm et al., 2022).

γH2AX and the DNA Damage Response (DDR): The presence of DSBs is marked by rapid phosphorylation of histone H2AX (γH2AX) (Kinner et al., 2008; Rahmanian et al., 2021; Prabhu et al., 2024). Although quantitative dose–response data in lung cancer are limited, the number of γH2AX foci may increase rapidly when a critical strain is passed *in vitro*, indicating that cellular repair capacity is being systematically exceeded.

8-OHdG as a Mutational Signature: Oxidative stress during mechanical load may result in large-scale accumulation of 8-hydroxy-2′-deoxyguanosine (8-OHdG) (AbdulSalam et al., 2016; Gorini et al., 2021; Hahm et al., 2022). In the lung TME, this specific lesion may act as a “molecular clock” of mechanochemical damage. It is well established that 8-OHdG lesions can cause G:C to T:A transversions, which promote oncogenic transformation and have a recognized role in lung carcinogenesis (Nakabeppu et al., 2006; Kryston et al., 2011). However, it is critical to distinguish between the accumulation of oxidative damage markers and the permanent fixation of specific driver mutations: while 8-OHdG is a validated pre-mutagenic lesion, evidence for direct deep-sequencing demonstrating that mechanical tension alone generates permanent, fixed driver mutations (e.g., in EGFR, KRAS, or TP53) in intact tissue remains a critical open gap.

#### 4.1.3 Ferroptosis: the direct mechanochemical link to lipid peroxidation

The most conclusive example of mechanochemical coupling in experimental systems is the relationship between mechanical strain and ferroptosis. This may not merely be an indirect effect but rather a direct conversion of physical force into a specific form of cell death, as demonstrated by experimental evidence in lung cells (Jiang et al., 2023).

Mechanical Accumulation of Lipid Peroxides: Experimental evidence clearly shows that mechanical stretch causes rapid accumulation of lipid peroxides in lung cell membranes. Physical stretching may increase the intermolecular gap between phospholipids and reduce steric hindrance for ROS-mediated hydrogen abstraction from polyunsaturated fatty acids (PUFAs) (Ngo et al., 2015; Yang et al., 2016).

The Chain Reaction: Lipid peroxidation is a self-propagating chain reaction that may be initiated by mechanical force, causing production of reactive aldehydes such as 4-hydroxynonenal (4-HNE) and malondialdehyde (MDA) (Yang et al., 2022).

4-HNE as a Genotoxic Agent: 4-HNE is relatively stable, unlike short-lived ROS, and can diffuse from the stretched plasma membrane into the nucleus. Upon entry, 4-HNE may react with deoxyguanosine to generate bulky DNA adducts (Feng et al., 2003; Zhong and Yin, 2015). These adducts may cause physical bending of the DNA double helix and amplify its susceptibility to mechanical forces passing through the nuclear lamina.

Ferroptotic Selective Pressure: High-intensity mechanical stress may result in terminal ferroptosis, whereas chronic sub-lethal stretch may exert selective pressure, driving expansion of cell populations that acquire resistance to high levels of lipid peroxide. These cells may accumulate high levels of mutations and develop features associated with persistent phenotypes that contribute to lung cancer initiation and therapeutic resistance (Konishi et al., 2023; Wang S.-A. et al., 2024).

#### 4.1.4 Nuclear envelope failure and cytoplasmic intrusion

The mechanical characteristics of a tumor (stiffness >15 kPa in experimental measurements) may lead to extreme nuclear shapes. In combination with high-magnitude cyclic stretch (as demonstrated to cause nuclear envelope damage *in vitro*), the nuclear envelope may reach its elastic breaking point, specifically the Lamin A/C scaffold (Denais and Lammerding, 2014; Denais et al., 2016).

Transient Nuclear Rupture: These ruptures may not be immediately fatal but may allow “unauthorized” exchange of material between the cytoplasm and nucleoplasm (Cho et al., 2019; Shah et al., 2021).

TREX1 Attack: The cytoplasmic exonuclease three-prime repair exonuclease 1 (TREX1) may infiltrate the nucleus during these rupture events. TREX1, a protein that normally eliminates viral DNA, may also target mechanically damaged genomic DNA, ultimately resulting in aggressive degradation of the cell’s own DNA and amplification of the DSB signal (Nader et al., 2021).

Lagging chromosomes may be captured in micronuclei under mechanical stress during mitosis. These micronuclei are very fragile and can burst open under tissue tension, causing catastrophic breakage and random reassembly of one chromosome (chromothripsis) (Hatch et al., 2013; Mohr et al., 2021). This phenomenon is one of the key reasons for swift genomic changes observed in the aggressive subtypes of lung cancer.

#### 4.1.5 Quantifying the pathological outcome

A pathological trajectory from physical stress to genomic collapse can be traced in experimental models. When stretch amplitude increases to pathological levels, chromosomal aberrations increase markedly, accompanied by a concomitant decline in DNA-replication fidelity (Wilhelm et al., 2020; Lai et al., 2022). Based on the current mechanistic evidence, it is inferred that such mechanical perturbations may lead to significant increases in chromosomal abnormality along with the reduced accuracy of replication (Uhl et al., 2011; Shaikh et al., 2022).

Moreover, the synergistic release of HMGB1 during such mechanochemical stress states has been observed to increase by approximately 13-fold in human lung microvascular endothelial cells subjected to 18% cyclic stretch for 24 h compared with statically cultured controls, representing a key alarmin that alerts the immune microenvironment to ongoing genomic collapse (Wolfson et al., 2014). This signaling pathway may also continue to drive the well-known “inflammatory–dysplastic–carcinoma” progression in lung tissue subjected to mechanical stress (Simi et al., 2015). Notably, this 13-fold increase in synergistic release of HMGB1 is obtained under defined experimental conditions including cell type, stretch duration, and detection assay; therefore, this value cannot be generalized for clinical settings without appropriate caveats.

### 4.2 Mechanical suppression of DNA repair machinery: the path to mutational permanence

Whether DNA damage is transient or becomes a permanent oncogenic mutation depends on the efficiency of the cell’s DDR (Klein and Glazer, 2010). Mechanochemical perturbation may not only cause DNA damage but also block intrinsic repair systems in cells within the lung TME (Deng et al., 2020). Based on the available mechanistic evidence, pathological mechanical tension, together with resulting oxidative storm, may converge to inhibit the Akt/Nrf2 signaling axis and other repair pathways, thereby creating conditions in which mechanochemically- induced genetic errors may evade correction and become embedded in the genome (Northcott et al., 2018; Romani et al., 2022). It is essential to distinguish, however, between repair inhibition (which increases the probability of mutation fixation) and the direct mechanical generation of fixed driver mutations, a distinction that remains empirically unresolved.

#### 4.2.1 DDR-Mechanical paradox: why repair fails under tension

A single DSB in DNA is sufficient to recruit sensors (such as the MRN complex) and transducers (ATM/ATR) that trigger a rapid response. However, a proposed “Mechanical Paradox” in the lung suggests that pathological cyclic stretch may both increase the number of DSBs and decrease the kinetics of repair (Nava et al., 2020). It has also been noted that ECM stiffness regulates DNA repair efficiency in a nonlinear fashion: stiffness in an optimum range promotes efficient repair, while excess stiffness (as found in the TME) inhibits homologous recombination and promotes error-prone pathways (Deng et al., 2020). This context-dependent effect points out that mechanics can either promote or inhibit repair, depending on the physical context and cell type, highlighting that further validation in lung cancer models is required.

Structural Impediment of the Repair Complex: Repair proteins assemble in functional complexes (foci) in a specific chromatin landscape. These repair complexes cannot be stably anchored to the chromatin because it is in the “mechanically unzipped” and relaxed state, as discussed in Section 4.1. Pathological strain causes the physical “pull” of the nuclear lamina to be a disruptive force that disturbs the molecular “stitching” of DNA ends (Pfeifer et al., 2018; Salvador Moreno et al., 2019).

Energy Demanding Process of DNA Repair: DNA repair is an energy-consuming process. As described in Section 3.2, loss of p120-catenin can trigger DRP1-mediated mitochondrial fragmentation, potentially creating a metabolic crisis. This ATP depletion may establish a prioritized energy budget for the cell, enabling survival at the expense of high-fidelity DNA repair mechanisms, which may result in a switch to error-prone repair pathways, such as non-homologous end joining (NHEJ) (Scully et al., 2019; Jing et al., 2020).

#### 4.2.2 Akt signaling maladaptation: from survival to genomic instability

Within the realm of mechanochemical coupling, Akt exhibits a biphasic and ultimately maladaptive response (Xu et al., 2012; Yu et al., 2022).

Acute Over-activation and Feedback Inhibition: Many studies have shown that initial mechanical stress can lead to Akt phosphorylation as an adaptive response for survival (Birukov, 2009; Case et al., 2011). However, the association of mechanical stress with Akt signaling is biphasic and context-dependent. Akt can be activated by moderate, brief mechanical perturbation as a protective survival mechanism; chronic pathological cyclic stretch can lead to persistent activation of Akt, triggering negative feedback loops and maladaptive signaling (Yu et al., 2022). Most observations are based on non-lung cell types, requiring confirmation in lung epithelial cancer models. The overactivation could make the cell insensitive to the specific Akt-signals that are needed to recruit DNA-PKcs (DNA-dependent protein kinase), a master regulator of NHEJ (Toulany et al., 2012).

The Akt-mTOR-ROS Loop: Mechanical strain may cause Akt activation, which triggers the mTOR pathway, which in turn leads to production of more ROS, which then acts as another mechanism to activate Akt. This mtROS may result in modification of Akt itself, particularly at cysteine residues, “freezing” the kinase in an inactive or faulty conformation (Ahmad et al., 2014; Su et al., 2019). In the absence of proper Akt signaling, cells with damaged DNA may not undergo the “arrest-and-repair” cycle and may enter mitosis prematurely.

#### 4.2.3 The Nrf2 conflict: the collapse of the antioxidant and repair shield

Nrf2 is perhaps one of the most important TFs involved in the maintenance of cellular redox and genomic integrity. However, emerging evidence indicates that under mechanochemical stress in lung cancer models, a critical failure may occur in the Nrf2 system (Satoh et al., 2016; Sánchez-Ortega et al., 2021).

The tipping point of Oxidative Overload: Although low levels of ROS activate Nrf2, it is proposed that the large, explosive NOX2-derived ROS burst produced by pathological cyclic stretch acts as a “chemical hammer.” This overload is believed to cause the carbonylation of Nrf2 and its chaperone Keap1, leading to the failure of Nrf2 to translocate into the nucleus (Brandes et al., 2014; Jiang et al., 2025). Importantly, the relationship between mechanical-oxidative stress and Nrf2-Akt signaling is biphasic and context-dependent. Moderate and transient mechanical perturbation triggers ROS as a protective adaptive response, inducing Nrf2 activation and subsequent upregulation of antioxidant enzymes and DNA repair genes. Conversely, chronic excessive mechanical stress and oxidative burden can ultimately breach this adaptive system, resulting in carbonylation-induced silencing of Nrf2 and maladaptive hyperactivation of Akt. In the lung TME, the exact timing and location that distinguish protective activation from pathological suppression remain poorly characterized and represent an important area for future investigation.

Suppression of Nrf2-Driven Repair Genes: Nrf2 regulates not only antioxidant enzymes but also transcription of key DNA repair genes, including *OGG1*, which repairs 8-OHdG lesions. Based on the current synthesis, in mechanically strained lung tissue, although levels of oxidative alarmins such as HMGB1 may be significantly elevated, the nuclear content of Nrf2 may be paradoxically reduced. This “Nrf2-silencing” under mechanical stress may be responsible for lack of repair of oxidative base damage (8-OHdG), which is associated with characteristic C>A transversions in aggressive lung malignancies (Vlahopoulos et al., 2019; Wang et al., 2025). It is critical to align the timing and context: acute mechanical stress may transiently activate Nrf2 as an adaptive response, whereas chronic, overwhelming oxidative load may exhaust and suppress this pathway.

Competing for co-factors: Nrf2-mediated antioxidant responses and DNA repair systems compete for the same pool of co-factors (NADPH and glutathione). Lipid peroxidation (the ferroptosis link) may constantly occur in the mechanochemical engine, potentially starving DNA repair enzymes of the reducing power they require to function (Hyun, 2020; Kasai et al., 2020).

#### 4.2.4 Mechanochemical inhibition of high-fidelity repair

Homologous recombination (HR) is the high-fidelity pathway, whereas NHEJ is the error-prone pathway in the hierarchy of DNA repair. The lung TME (characterized by increased stiffness and cyclic stretch in experimental models) is hypothesized to actively promote a “Pathway Switch” from HR to NHEJ (Jiang Y. et al., 2022). This hypothesis is supported by studies showing that excessive ECM stiffness can suppress homologous recombination in some cell types, though direct evidence in lung cancer models remains limited (Deng et al., 2020).

Modification of Histone State by Mechanical Tension: Mechanical tension may cause certain histone modifications that regulate silencing of HR-related genes such as *RAD51*. This “mechanical epigenetic” remodeling may ensure that even if the cell attempts repair, it will instead use the low-fidelity NHEJ pathway, which is often associated with small insertions, deletions, and translocations (Pai et al., 2014; Misova et al., 2021).

The 4-HNE generated during mechanochemically induced lipid peroxidation may form direct adducts at the active sites of DNA polymerases and ligases. Such chemical modification can impair the function of repair machinery, leading to persistent retention of DNA adducts and subsequent fixation of mutations in the next round of DNA replication (Feng et al., 2004).

#### 4.2.5 Clonal selection and the conditions favoring mutational fixation

This mechanochemical suppression may culminate in damage accumulation and clonal evolution. Cells that survive pathological cyclic stretch and oxidative storm may “accept” genomic damage (Broders-Bondon et al., 2018; Frankell et al., 2023).

Fixation of the HMGB1 Signal: Substantial release of HMGB1, acting as a paracrine signal, may promote survival of mutation-bearing “persister” cells. The mechanochemical engine may prevent cells with damaged genomes from undergoing apoptosis, at least in part due to activation of the MDM2 pathway by Akt (He et al., 2018; Chibaya et al., 2021).

Path to permanent mutation: When an 8-OHdG or SSB lesion is passed on to a daughter cell after a cell division or to the next cell in the line of descent, the lesion is at risk of becoming a permanent base-pair substitution or chromosome rearrangement. At this “Point of No Return” in lung cancer evolution, the stressed tissue may eventually give rise to a genetically mutated malignancy—only a few cell divisions away (Amente et al., 2019; Gorini et al., 2021). It must be reiterated, however, that this trajectory represents a plausible mechanistic model: direct demonstration that mechanical force alone drives permanent fixation of specific driver mutations in intact lung tissue requires spatial multi-omics and deep-sequencing validation.

Critical evidence gap: It is essential to distinguish between the well-documented ability of mechanical stress to induce transient DNA lesions (e.g., γH2AX foci and 8-OHdG) and the fixed genomic alterations that constitute cancer driver mutations. While the above model proposes that mechanochemical stress creates a mutator phenotype that may facilitate the eventual fixation of mutations, direct evidence linking specific mechanical thresholds to the generation of canonical driver mutations in native lung tissue remains a critical unmet need in the field (Frankell et al., 2023). Thus, we present this sequence as a working hypothesis requiring direct experimental validation rather than an established pathway. The full hypothesized progression from mechanochemical injury to malignant phenotype is schematized in Figure 3.

**FIGURE 3:**
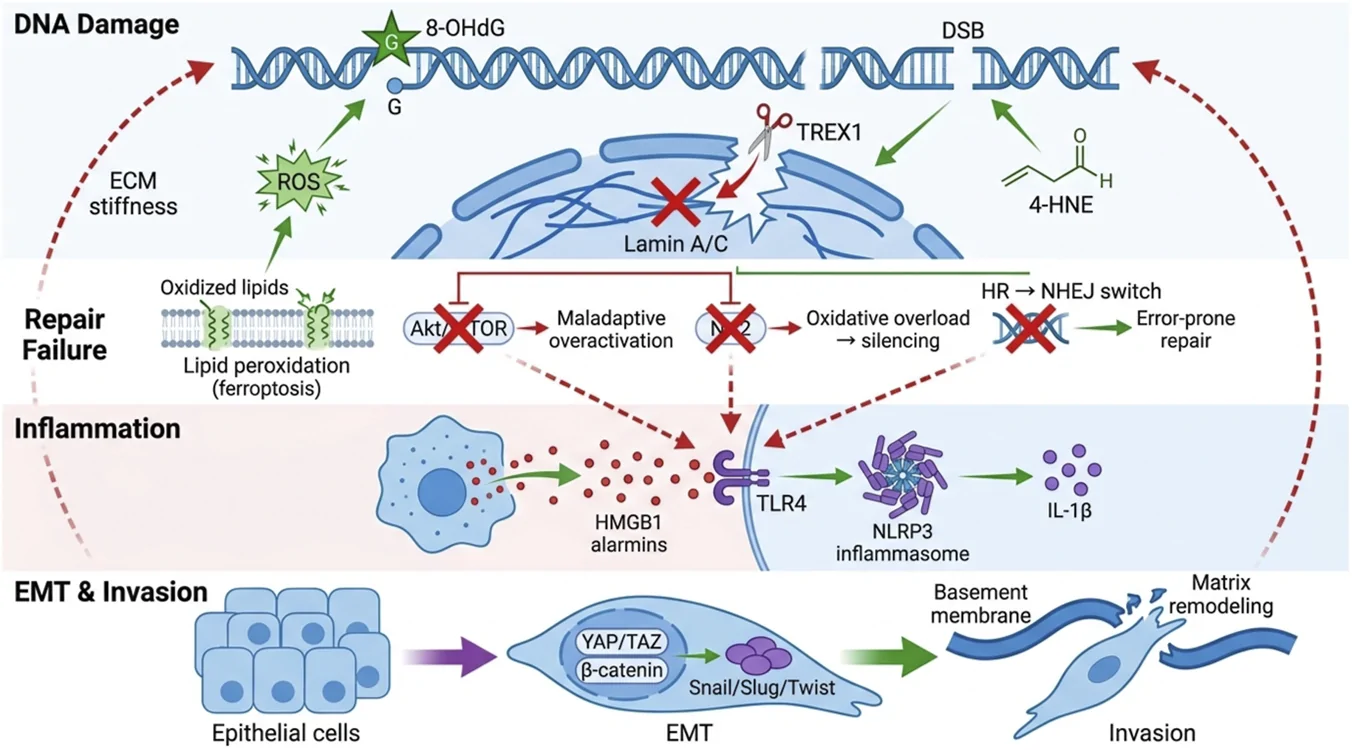
Genomic instability and malignant transformation.

## 5 Malignant transformation: microenvironment and phenotypic switching

A permissive and supportive environment is required for a mutation-bearing cell to evolve into a fully established clinical malignancy. In lung cancer, this niche is not a silent bystander but may be “mechanochemically primed” to actively support and promote disease development. The subsequent steps—leading to a local inflammatory storm and induction of a senescence-associated secretory phenotype (SASP)—are discussed here as proposed consequences of mechanochemical stress, potentially converting lung tissue into a pro-tumorigenic microenvironment (Campisi, 2008; Chen et al., 2025).

### 5.1 The inflammatory storm and SASP: the pro-oncogenic feedback

The main characteristic of the final step of the proposed mechanochemical engine is translation of intracellular oxidative stress to extracellular inflammatory signals. The process may be characterized by release of danger-associated molecular patterns (DAMPs) and induction of cellular senescence, potentially leading to a vicious cycle of tissue remodeling and malignant selection (Malaquin et al., 2016; Zhang F. et al., 2024).

#### 5.1.1 The HMGB1 burst: alarmin signaling under mechanical stress

HMGB1 is a non-histone nuclear protein that acts as a DNA chaperone. It is translocated to the extracellular space to become a potent alarmin. In lung mechanobiology settings, strong HMGB1 release may indicate failure of tissue homeostatic machinery (Klune et al., 2008; Andersson et al., 2018).

Oxidative stress alone or mechanical (cyclic) strain alone may cause moderate HMGB1 leakage, whereas their combination may result in synergistic elevation in experimental systems. This marked upregulation may represent an important threshold event that, together with disrupted nuclear envelope integrity and elevated plasma membrane permeability, results in high levels of alarmin secretion (Wolfson et al., 2014; Yu et al., 2015).

Pathological Mechanical Tension: HMGB1 may not be simply passively released from dead cells. Regulated active secretory pathways can be activated by the mechanochemical cascade. Acetylation of HMGB1 by ROS may impede its re-entry into the nucleus and, therefore, facilitate its accumulation in the cytoplasm, where it is secreted in vesicles. This mechanism may maintain high extracellular HMGB1 even under sublethal mechanical stress conditions (Erlandsson Harris and Andersson, 2004; Wang Y. et al., 2019). Extracellular HMGB1 may bind to neighboring epithelial and immune cells via RAGE and TLR4. This interaction may trigger a paracrine signaling cascade that extends the oxidative stress phenotype throughout the local tissue and affects healthy bystander cells (Scaffidi et al., 2002; Deng et al., 2013).

#### 5.1.2 NLRP3 inflammasome activation: the “mechanical” inflammatory engine

Robust release of HMGB1 may be directly linked to assembly of the NLRP3 inflammasome, a multiprotein complex that acts as a hub for the inflammatory storm (Frank et al., 2015).

The Two-Hit Mechanical Model: Mechanochemical coupling is hypothesized to be the source of both signals needed to activate NLRP3. Activation of TLR4 by binding with HMGB1 and mechanosensitive signaling pathways may provide “Signal 1” (priming). It is proposed that “Signal 2” (activation) may arise from mtROS production in response to DRP1-induced mitochondrial fragmentation and pathological mechanical stretch (Trachalaki et al., 2021; Jiang H. et al., 2022).

The activated NLRP3 inflammasome cleaves pro-interleukin-1β (pro-IL-1β) and pro-interleukin-18 (pro-IL-18) to generate their active forms. IL-1β is also a major player in the lung microenvironment, acting on fibroblasts and matrix stiffness in ways that may influence the desmoplastic reaction (Mia et al., 2014; Gairola et al., 2024). This may create a feedback loop: mechanical stress induces HMGB1/NLRP3 expression, which generates IL-1β that drives matrix remodeling. This remodeling alters tissue stiffness, thereby modulating mechanical stress and perpetuating the cycle (Mancini et al., 2024).

The NLRP3 axis may provide a continuous extrinsic ROS source for the chronic inflammatory environment. Together with decreased repair mechanisms, this may favor stalling of oncogenic mutations and, thus, increase the pro-tumorigenic niche (Sharma and Kanneganti, 2021; Wang et al., 2023).

#### 5.1.3 Mechanically induced senescence: the SASP program

The “physical stage” may exert strong control on the cell fate through induction of cellular senescence. Pathological mechanical stretch may induce senescence when lung epithelial cells fail to repair DNA damage (Du et al., 2025; Martín-Vicente et al., 2025).

Stiffness-Senescence Link: Chronic exposure to a stiffened ECM is a powerful pro-senescence signal in experimental models. Senescent cells are characterized by flattened and enlarged morphology and are known to express p16INK4a and p21 as a result of high intracellular tension (Blokland et al., 2022; Song et al., 2026).

Senescent lung cells are not metabolically silent but may acquire an SASP. This secretome contains high concentrations of proinflammatory cytokines (IL-6 and IL-8), growth factors (VEGF), and matrix metalloproteinases (MMPs), similar to those reported to be enriched in the SASP of lung senescence (Maciel-Barón et al., 2016; Chen et al., 2025).

MMPs and Matrix Remodeling: The MMPs secreted as part of the SASP may be mechanically activated, with MMP-2 and MMP-9 playing vital roles in ECM remodeling in lung pathology. They may not restore health; rather, they may destroy the basement membrane and remodel the ECM to provide “tracks” for future tumor cell migration. The SASP may promote reconfiguration of the lung niche from a structured, protective barrier to an open porous niche that favors invasion (Cai et al., 2023; Wolosowicz et al., 2024).

#### 5.1.4 The immunoevasive consequence of the storm

The final stage of the “inflammatory storm” in the evolution to malignancy is the development of an “immunosuppressive microenvironment.”

Enhanced release of HMGB1 and SASP factors may specifically recruit myeloid-derived suppressor cells (MDSCs) and M2-type macrophages. These cells may inhibit function of T-cells and natural killer (NK) cells that would otherwise kill mutated epithelial cells (Jin et al., 2020).

Tissue stiffening and solid stress associated with this inflammation may physically limit immune cell entry into the “mechanochemical core,” thus creating a physical barrier for the developing tumor. This is consistent with recently published studies demonstrating a role for ECM stiffness in immune exclusion (Yu et al., 2024; Zhang and Zhang, 2025).

#### 5.1.5 Quantifying the niche: a summary of evidence

The process of establishing this malignant niche may be measurable in experimental systems. Chronic mechanical stretch regions in lung models have been reported to exhibit higher levels of SASP markers and higher levels of extracellular HMGB1, consistent with other mechanotransduction studies of lung pre-cancer niche formation (Rapoport et al., 2020; Du et al., 2025). This section further demonstrates that the mechanochemical engine may not only operate at the single-cell level but also reshape entire tissue ecology, creating conditions in which mutations may evolve into invasive carcinoma (Northcott et al., 2018).

### 5.2 Physical barrier collapse and EMT: from structural failure to invasive signaling

For a localized genomic instability to progress to systemic malignancy, physical barriers must be breached. The alveolar–capillary barrier in the healthy lung is held together by a complex system of intercellular junctions that create an apical–basal polarity and limit paracellular permeability (Nelson, 2009; Yu and Elble, 2016). However, these junctions are not merely structural; they are also tightly linked to the initiation of the EMT process, a phenotypic change that involves the loss of these junctions (Heise et al., 2011; Khalili-Tanha et al., 2025).

#### 5.2.1 The tight junction: ZO-1 and claudins are vulnerable

The most apical components of the junctional complex are tight junctions (TJs), which contain transmembrane proteins such as claudins and peripheral membrane proteins such as ZO-1 (Otani and Furuse, 2020).

The ROS burst produced by mechanical activation of NOX2 and dysfunctional mitochondria may affect ZO-1 and claudins and has been linked to breakdown of intermolecular interactions between ZO-1 and the actin cytoskeleton (Inumaru et al., 2009; Shen et al., 2024).

Mechanical Dissociation: Cyclic mechanical stretching applies physical force to the plasma membrane, thereby regulating the TJ complex. The binding of claudins to the scaffolding protein ZO-1 is substantially decreased in the presence of ROS, leading to endocytosis of junctional proteins. This exposes the paracellular space and disrupts the cell–cell interface (Spadaro et al., 2017; Stamatovic et al., 2017).

Loss of “Gate and Fence” Functions: This loss of polarity may be essential for EMT and may allow pro-tumorigenic receptors (such as growth factor receptors and integrins) to be distributed across the entire cell surface (Kyuno et al., 2021; Kim et al., 2023).

#### 5.2.2 The junction-to-nucleus signaling: unleashing the EMT transcriptome

Loss of TJs may not be merely a structural event; it may also be a signaling event. This process is known as “junction-to-nucleus signaling” (Gonzalez and Medici, 2014).

ZONAB and Cell Cycle Progression: Normally, ZO-1-associated nucleic acid-binding protein (ZONAB) is sequestered at the plasma membrane by ZO-1. Evidence suggests that ZO proteins redundantly retain ZONAB at TJs. Upon degradation and internalization of ZO-1 by mechanochemical stress, ZONAB may move to the nucleus. In the nucleus, ZONAB may activate the expression of PCNA and cyclin D1, thus pushing the cell out of homeostatic quiescence and into the proliferating mode, which may allow fixation of mutations (Balda et al., 2003; Sourisseau et al., 2006; Lima et al., 2010).

β-catenin release and Wnt synergy: Similarly, rupture of adherens junctions (secondary to TJ breakage) may release β-catenin from the membrane pool. Without p120-catenin, β-catenin levels may not be maintained, and it can translocate to the nucleus where it acts as a co-activator for TCF/LEF TFs, thus potentially upregulating the EMT program by upregulating the expressions of Snail, Slug, and Twist (Medici et al., 2008).

#### 5.2.3 Mechanical induction of EMT transcription factors

Pathological tension (increased matrix stiffness and cyclic stretch in experimental models) may be a direct inducer of the EMT transcriptome via several mechanosensitive pathways (Dupont et al., 2011).

YAP/TAZ as Mechanochemical Rheostats: High matrix stiffness favors Yes-associated protein (YAP) and TAZ nuclear translocation (Dupont et al., 2011). These proteins bind to TEAD TFs to stimulate a mesenchymal phenotype. Importantly, the oxidative environment (ROS) is believed to enhance YAP activity, potentially leading to a synergistic effect between “force” and “chemistry” which “locks” the cell in a mesenchymal state (Amanda et al., 2024).

TGF-β Activation via Force: TGF-β is present in the lung ECM bound to the Large Latent Complex (LLC). Activation of TGF-β may occur in response to mechanical tension without proteolysis, as it is in the LLC (Buscemi et al., 2011). This mechanically released TGF-β may then act in an autocrine manner to stimulate the expression of mesenchymal markers such as vimentin and N-cadherin and to continue downregulation of E-cadherin expression (Saito and Nagase, 2015).

#### 5.2.4 ROS-driven feedback in EMT: the Snail-NOX2 loop

There may be a bidirectional relationship between mechanochemical stress and EMT. In addition to inducing EMT, stress may further fuel the oxidative engine, creating a positive feedback loop that has been broadly substantiated by experiments (Barnett et al., 2011).

Snail-Mediated NOX Activation: The EMT TF Snail has been demonstrated to activate the expression of NOX subunits, increasing the cell’s ability to produce ROS during EMT (Kim and Cho, 2014).

Mitochondrial Re-programming: EMT may be accompanied by a metabolic switch to glycolysis and increased generation of mitochondrial ROS, which is required to fuel the high energy needs for cell migration. This metabolic switch may guarantee that, once the EMT switch is activated by mechanical stress, the cell can experience chronic oxidative stress that further perpetuates genomic instability and phenotypic plasticity (Lee et al., 2019).

#### 5.2.5 Consequences for invasion: the pathological opening

The final consequence of the ZO-1/claudin breakdown and EMT is the gain of an invasive phenotype.

Proteolytic Remodeling: When cells become mesenchymal-like in lung cancer, they may release large amounts of MMPs (particularly MMP-2 and MMP-9). These enzymes may be physically stimulated by the acidic and oxidative microenvironment of the tumor (Sutoo et al., 2020). They may break down the basement membrane, allowing the cell to break away from the primary tumor—a process supported by evidence that MMPs can degrade basement membranes and promote ECM turnover in disease states (Ashja Ardalan et al., 2024; Khalili-Tanha et al., 2025).

The “Leader-Follower” Dynamic: In the lung, a “leader” cell that has acquired mesenchymal properties may pull a “follower” epithelial cell through a remodeled ECM. Stiffness and tension gradients within the TME may drive this collective migration. Studies indicate that stiff collagen stimulates invasive behavior in tumor cells and that leader cells remodel the ECM to facilitate follower cell invasion (Carey et al., 2013; Lichtenberg et al., 2024).

(This conceptual schematic illustrates the proposed cascade from mechanochemical stress to genetic destruction and phenotypic switching. ROS and 4-HNE may directly attack DNA, generating 8-OHdG adducts and DSBs. Concurrent nuclear envelope rupture may allow TREX1 nuclease infiltration. DNA repair may be suppressed through three proposed mechanisms: Akt/mTOR maladaptive overactivation, Nrf2 silencing, and HR-to-NHEJ pathway switching. Damaged cells may release HMGB1, potentially activating the NLRP3 inflammasome and IL-1β production. These insults may drive EMT via YAP/TAZ and β-catenin-mediated upregulation of Snail/Slug/Twist, leading to invasion and matrix remodeling that feeds back to increase stiffness. Green indicates damage; red indicates inhibition/pathology; purple indicates inflammation/EMT. This figure represents a conceptual pathway synthesis rather than plotted experimental datasets.)

## 6 Therapeutic perspectives: toward mechanopharmacology

### 6.1 Precision antioxidants and growth factors: restoring genomic integrity

The discovery of the proposed mechanochemical engine has opened the era of “primordial prevention” and “mechanoprotection.” By employing growth factor-mediated repair and Nrf2 activators, the transition from mechanical strain to genetic collapse could theoretically be interrupted (Gargalionis et al., 2024; Stewart and Gao, 2026).

#### 6.1.1 Nrf2-Keap1 rheostat: molecular redesign under stress

The cell’s most powerful defense system against oxidative bursts is the Nrf2 system. In many cases, the endogenous Nrf2 response may be short-circuited by excessive ROS levels under high-magnitude stretch; however, cyclic stretch has also been shown to promote Nrf2-ARE activation in lung cells in certain contexts (Papaiahgari et al., 2007; Wang G. et al., 2020). The vision of mechanopharmacology is to achieve robust Nrf2 activation with specific polyphenolic activators that have been shown to play a central role in protection from oxidative stress through the Keap1-Nrf2 pathway (Baird and Yamamoto, 2020).

Resveratrol: Beyond Scavenging to Covalent Modification: Resveratrol is often described solely as an antioxidant, but this may be an oversimplification. Its true significance for the lung has been attributed to its capacity to dissociate Keap1 from Nrf2, disrupting their binding and impairing Nrf2 ubiquitination (Hao et al., 2020). Although it is not explicitly stated that resveratrol modifies the specific cysteine residues in Keap1 (Cys151, Cys273, and Cys288), these cysteines have been shown to be essential for Nrf2 regulation (Chi et al., 2024).

Quercetin and the OGG1 Repair Axis: Unlike most other antioxidants that target lipid peroxidation, quercetin may improve Base Excision Repair (BER). The enzyme OGG1 is needed for repair of 8-OHdG, which is a hallmark of mechanochemical DNA damage, and quercetin has been found to inhibit oxidative DNA damage and modulate DNA repair processes through Nrf2 signaling (Weiss-Sadan et al., 2023; Chen et al., 2024). Quercetin has been reported as a transcriptional inducer of OGG1 by binding to the ARE in the promoter of the *OGG1* gene, a finding consistent with reported activation of Nrf2 and regulation of genes involved in DNA repair by quercetin (Moratilla-Rivera et al., 2023).

A critical hurdle for Nrf2 therapy is systemic bioavailability. Paradoxically, increased vascular permeability associated with eNOS uncoupling and the surge in HMGB1 release may promote local accumulation of these hydrophobic polyphenols at sites of mechanical stress, specifically the “invasive front” within the lung. This aligns with the overall function of Nrf2 activators in targeting stress-prone tissues (Cho et al., 2006). However, it must be emphasized that generic, untargeted antioxidants have consistently failed in clinical settings because they cannot accumulate at localized, organelle-specific high-tension microdomains. The necessity for localized, 3D/nanoparticle-targeted delivery vehicles should be emphasized in future mechanopharmacological designs.

#### 6.1.2 FGF-10: the biophysical antidote to structural collapse

While Nrf2 activators provide chemical protection, fibroblast growth factor-10 (FGF-10) may restore structural integrity. To support the lung’s epithelial identity and prevent phenotypic switching associated with EMT, FGF-10 is a key paracrine factor well established in lung epithelial development and regeneration (Itoh, 2016; Yuan et al., 2018).

Loss of p120-catenin acts as a “gatekeeper” switch for mitochondrial ROS production. FGF-10 signaling via its high-affinity receptor fibroblast growth factor receptor 2b (FGFR2b) activates the PI3K/Akt pathway in a controlled, physiological manner (as opposed to pathological Akt over-activation), a pathway known to be a downstream target of FGF10-FGFR2b signaling (Yin and Ornitz, 2020). This represents a physiological form of Akt activation that leads to phosphorylation and membrane stabilization of E-cadherin and p120-catenin, findings consistent with the known role of FGF10 in regulating epithelial cell adhesion (Carraro et al., 2009). FGF-10 physically blocks the “leak” of pro-oxidative signals from the plasma membrane to the mitochondria by strengthening the adherens junctions.

Mitochondrial Re-Coupling and DRP1 Inhibition: Regarding the mitochondria, FGF-10 has been reported to reverse pathological stretch-induced mitochondrial fragmentation and to prevent the development of ROS accumulation and oxidative stress, supporting the re-coupling effect of FGF-10 and inhibition of DRP1 (Liu L. et al., 2022; Lin L. et al., 2024). FGF-10 may prevent DRP1-mediated mitochondrial fragmentation and help maintain mitochondrial membrane potential (ΔΨm), thereby reducing mtROS leak, a phenomenon known to increase with DRP1-dependent fragmentation (Cho and Kim, 2024). This results in “mitochondrial resilience,” which allows the organelle to generate ATP for DNA repair without inflicting oxidative damage to the oxidative machinery, similar to the effect of FGF-10 in preventing mechanical stretch-induced DNA damage (Upadhyay et al., 2005).

FGF-10 may also suppress the NLRP3 inflammasome, representing a key mechanism by which FGF-10 reduces inflammatory responses, with direct clinical relevance given the surge in HMGB1 levels observed under mechanochemical stress (Kong et al., 2022; Huang et al., 2023). FGF-10 may protect the integrity of the cell and nucleus, potentially by stimulating the expression of Lamin A/C, and may prevent catastrophic release of HMGB1 (Salminen, 2026). FGF-10-treated cells may have a much smaller “inflammatory footprint” and may not recruit immunosuppressive cells that would otherwise protect the developing tumor.

#### 6.1.3 Synergy: the integrated mechanochemical defense

The synergy between chemical defense and structural repair, which is mediated by Nrf2 and FGF-10, respectively, may represent a key to future lung cancer prevention, consistent with the individual protective roles of each pathway in preclinical models (Satoh et al., 2016). However, the historical failure of broad-spectrum systemic antioxidants in cancer prevention trials underscores that effective “mechanoprotection” requires more than just scavenging ROS. It necessitates the strategic delivery of such agents to specific organelles (mitochondria) or high-tension microdomains, a challenge that current pharmacokinetic approaches have yet to overcome.

The “Double-Lock” Mechanism: Quercetin/resveratrol may “lock” DNA in a repair-ready state, while FGF-10 may “lock” cellular junctions and mitochondria in stable structure. This dual intervention is hypothesized to uncouple high-magnitude stretch from its downstream oncogenic effects as Nrf2 activators have chemopreventive effects and FGF-10 protects against mechanical stretch-induced DNA damage (Upadhyay et al., 2004; Malhotra et al., 2014).

These interventions may be best targeted to the “mechanochemical window”: the period from when tissue stiffness starts to increase (10–15 kPa) to the point when DNA repair mechanisms have been permanently epigenetically silenced. This timing aligns with the discovery of a modest supra-physiological stiffness window that allows tumor-initiating conversion, confirming the relevance of an interventional window (Panciera et al., 2020). During this window, FGF-10 and Nrf2 activators may reverse the “oxidative scar” arising from chronic mechanical stress, thereby restoring the physiological elasticity and genomic stability of the lung, interventions that have been shown to reduce oxidative stress and structural damage (Cho et al., 2006; Jiang T. et al., 2022).

#### 6.1.4 Quantitative clinical implications

The co-administration of Nrf2 activators and FGF-10 is hypothesized to yield a marked reduction in γH2AX foci (DSBs) and normalization of HMGB1 surge, an extrapolation consistent with the known protective effects of both pathways in lung injury (Huang et al., 2023; Yousefi Zardak et al., 2024). Based on these predictions, mechanopharmacology may be not only a theoretical possibility but also a viable therapeutic approach that could change the mutational course of lung cancer in high-risk populations. Evidence suggests that activation of Nrf2 and administration of FGF-10 reduce oxidative stress and DNA damage associated with lung carcinogenesis (Tao et al., 2018; Settayanon et al., 2025).

### 6.2 Physical interventions: modulating the Piezo1 channel and ECM stiffness

The oxidative engine may be the “chemical smoke” of lung cancer, and physical deformation of the cell may be the “fire.” To address the source, cellular perception and response to force must be directly modulated. Increasing evidence indicates that inhibiting mechanosensing pathway components can disrupt pro-tumor signaling (Jiang Y. et al., 2022; Beyranvand, 2026). This section dissects the potential for targeting Piezo1 mechanosensors and ECM architecture as a way of “physical de-escalation,” consistent with the role of Piezo1 and ECM stiffness in tumor progression (Chen et al., 2018; Ibarrola et al., 2024).

#### 6.2.1 Piezo1 antagonism: silencing the mechanical “first responder”

Piezo1 is a nonselective cation channel with a propeller-like structure that detects nanonewton-scale changes in membrane tension (De Vecchis et al., 2021; Liu et al., 2025). Piezo1 may be the main conduit converting kinetic energy into pathological calcium (Ca^2+^) influx at high-magnitude cyclic stretch thresholds, a mechanism consistent with its established function in transducing mechanical stress into Ca^2+^ influx (Swain and Liddle, 2021; Wang et al., 2021).

Pathological Ca^2+^ influx via Piezo1 may directly activate CaMKII, leading to phosphorylation of the p47phox subunit of NOX2, extending known connections between Piezo1 activation, CaMKII activation, and oxidative stress (Liu H. et al., 2022; Lan et al., 2024). The peptide GsMTx4 and new small-molecule inhibitors of Piezo1 may block this “priming” of the NOX2 burst (Bae et al., 2011). This represents a conceptual “pre-emptive strike” against the oxidative engine.

The Nuclear Shield Effect: Piezo1 activation has also been linked to phosphorylation-dependent softening of the Lamin A/C network, consistent with evidence that Piezo1 activation influences Ca^2+^-dependent nuclear mechanics (Garcia-Castorena et al., 2025). Pharmacological inhibition of Piezo1 may prevent this nuclear weakening, thereby preserving genome stability even under extreme stretch, acting as a “Faraday cage” that shields the genome.

Targeting Piezo1 in the TME: Piezo1 is overexpressed in mechanically stressed tissue, and its inhibition would have a high degree of selectivity, targeting the “mechanically active” tumor front, while leaving the relatively quiescent healthy parenchyma intact. However, systemic inhibition of Piezo1 presents formidable safety challenges as Piezo1 is crucial for erythrocyte volume regulation and vascular baroreception; global blockade could induce severe cardiovascular complications. Therefore, clinical translation of this strategy would likely require localized delivery approaches rather than systemic administration. This aligns with Piezo1 overexpression in the TME and its potential as a tissue-selective therapeutic target (Thien et al., 2024; Zhang et al., 2026).

#### 6.2.2 Softening the stage: LOX inhibition and matrix remodeling

Cyclic strain exerts a force-multiplying effect, amplified by static resistance of a stiffened ECM (15–50 kPa in experimental measurements). Reduction of this “mechanical preload” may be key to restoring homeostatic mechanotransduction, supported by evidence that inhibition of the LOX family can reverse pathological ECM stiffening (Mammoto et al., 2013; Vallet and Ricard-Blum, 2019).

LOX-Mediated Cross-linking as a Target: Lysyl Oxidase (LOX) catalyzes oxidative deamination of lysine residues in collagen and elastin, which are responsible for covalent cross-links that lead to tissue stiffening (Gao et al., 2010; Vallet and Ricard-Blum, 2019). Small-molecule inhibitors such as β-aminopropionitrile (BAPN) or more sophisticated LOXL2-specific antibodies have been found to “de-stiffen” lung stroma, and LOXL2-selective inhibitors have been found to lower matrix stiffness in a dose-dependent manner in pulmonary fibrosis models (Jones et al., 2018). However, systemic LOX inhibition risks compromising arterial integrity and causing aortic aneurysms as LOX is essential for vascular wall maintenance. Thus, localized delivery strategies are essential for clinical translation of this approach. These agents reduce the elastic modulus of the TME, thereby imparting significantly higher “mechanical compliance” to the tissue, with 20% external stretch resulting in much lower, nonpathological internal cell stretch, as has been observed by LOX inhibition to reduce tumor stiffness and normalize mechanotransduction (Nicolas-Boluda et al., 2021; Setargew et al., 2021).

Breaking the Stiffness–EMT Loop: As described in Section 5.2, matrix stiffness is one of the main factors contributing to EMT via YAP/TAZ. “Softening” of the matrix through LOX inhibition, or use of PEGylated hyaluronidase (PEGPH20), may effectively degrade the physical scaffold that allows EMT transition (Deng et al., 2021). This will provide a “structural reset” in addition to the chemical protection known to be provided by Nrf2 activators, which is supported by preclinical studies where LOX inhibition has been shown to impair EMT and malignancy in lung cancer (Gao et al., 2010; Xiao and Ge, 2012).

#### 6.2.3 Nuclear-targeted mechanoprotection: the role of lamin stabilizers

The last frontier of physical intervention may be the nucleus itself. The therapeutic goal is to stabilize the nuclear envelope as nuclear rupture may be the “point of no return” for TREX1-mediated DNA attack and chromothripsis (Cho et al., 2017).

Retinoic Acid and Lamin A/C Upregulation: It is plausible that all-trans retinoic acid (ATRA) might upregulate Lamin A/C expression, resulting in increased mechanical “stiffness” (resilience) of the nuclear envelope as Lamin A/C upregulation has been observed with mechanical tension (Schuldt, 2013; Srivastava et al., 2021). In a “Mechanopharmacological” concept, ATRA might serve as a nuclear reinforcement agent, preventing transient ruptures that happen during 20% stretch, and protect the DNA from the “oxidative storm” in the cytoplasm, consistent with the well-established nuclear protective role of lamin A/C during mechanical stress (Kim J.-K. et al., 2017; Wang et al., 2022).

#### 6.2.4 Integrated strategies: the “Triple-Barrier” framework

These physical interventions combine to form a “Triple-Barrier” therapeutic approach:

The Membrane Barrier: Piezo1 inhibitors to block the very first mechanosensitive influx of calcium/ROS (Zhao et al., 2022; Wang et al., 2026).

The Stromatic Barrier: LOX inhibitors to make the TME less stiff and mechanically preloaded (Gao et al., 2010; Zhu et al., 2023).

The Nuclear Barrier: Lamin stabilizers such as ATRA to reinforce nuclear envelope integrity and prevent envelope rupture (Stefanello et al., 2021; Schultz et al., 2025).

Together with biochemical priming (Nrf2/FGF-10), a complete “mechanoprotective” shield could be developed. At the tissue level, this integrated strategy may tackle all aspects of the proposed mechanochemical engine, spanning from macro-scale tissue architecture to nano-scale DNA backbone, and adopts a holistic approach to lung cancer prevention and treatment. This aligns with the emerging principles of mechanomedicine (Gargalionis et al., 2024; 2025). Figure 4 presents an integrated framework of biochemical and physical interventions targeting the mechanochemical engine, summarizing the multilayered therapeutic strategies discussed above.

**FIGURE 4:**
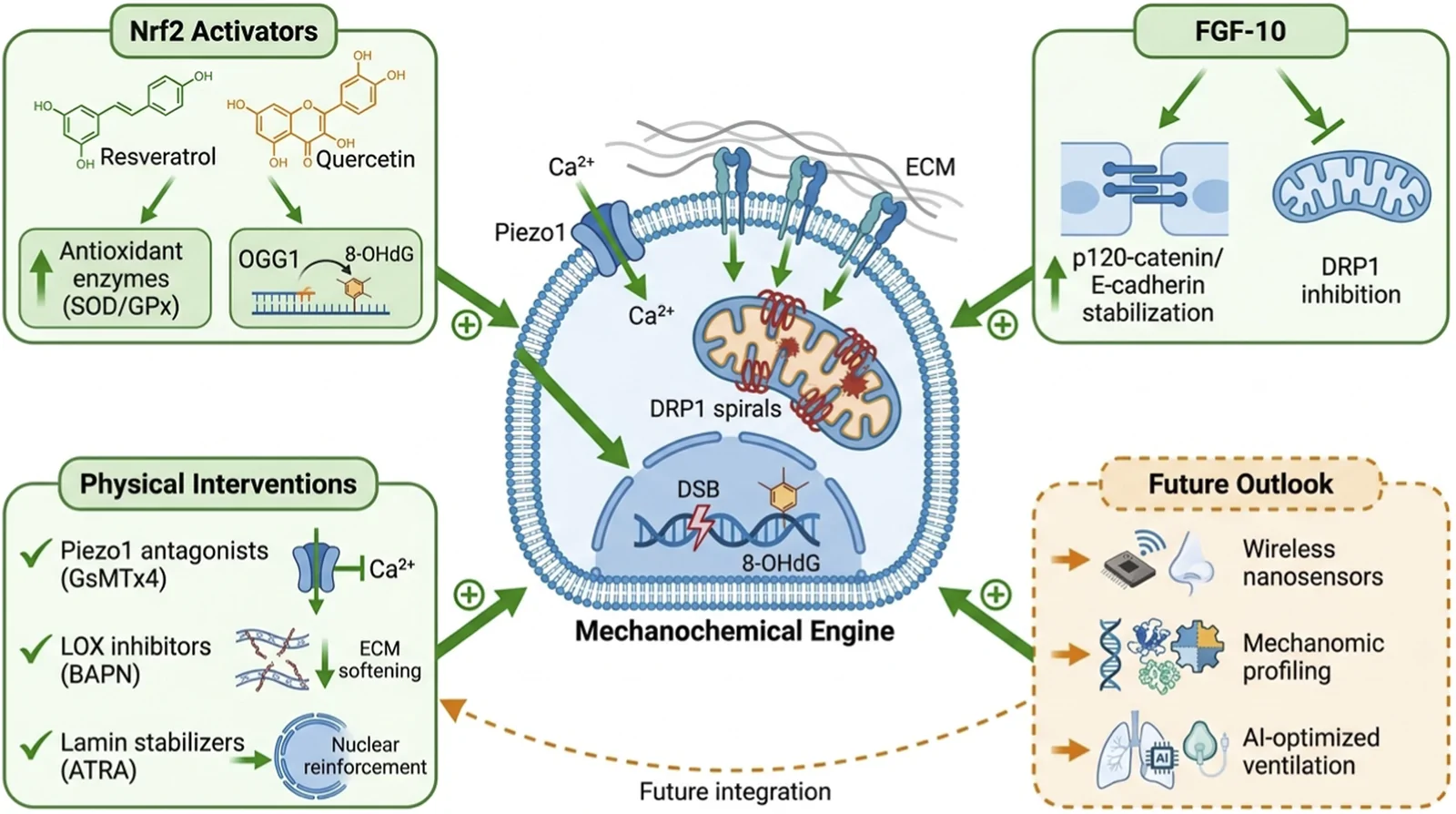
Therapeutic strategies and mechanopharmacology.

(This figure presents an integrated framework of biochemical and physical interventions targeting the mechanochemical engine. Nrf2 activators (resveratrol/quercetin) upregulate antioxidant enzymes and OGG1-mediated DNA repair. FGF-10 stabilizes p120-catenin/E-cadherin junctions and inhibits DRP1-mediated mitochondrial fission. Physical interventions include Piezo1 antagonists (GsMTx4), LOX inhibitors (BAPN) for ECM softening, and lamin stabilizers (ATRA). Future outlook technologies—wireless nanosensors, mechanomic profiling, and AI-optimized ventilation—promise real-time mechanical monitoring and personalized intervention. Green indicates therapeutic/protective; blue indicates cellular structures; orange indicates future technologies).

### 6.3 From hypothesis to therapy: the translational gap

The therapeutic strategies discussed above are supported by preclinical evidence but remain largely at the hypothesis-generating stage. Critical translational gaps must be addressed before clinical application. We present these concepts as a theoretical roadmap to stimulate discussion, not as near-term clinical realities.

## 7 Conclusion and future outlook: toward a mechanistic synthesis

The proposed mechanochemical engine in lung cancer represents a conceptual watershed in understanding oncogenesis. We may no longer be dealing with a purely stochastic model of genetic mutation but rather with a deterministic framework in which the chemical fate of the cell may be influenced by the physical microenvironment (Yuan et al., 2024). By systematically mapping the proposed trajectory from high-magnitude cyclic stretch experimental models to conditions favoring mutation fixation, this review has identified a set of mechanochemical “checkpoints” that may define how mechanical cues drive lung cancer progression—consistent with recent findings that mechanosignals and associated checkpoints may control this process (Katira et al., 2013; Shim et al., 2026).

### 7.1 The bridge function of mechanochemical coupling

The central theme of this work is that the lung TME may not be a passive background of cancer cells; it may be an active player in the disease process. The proposed mechanochemical coupling may take place through three core transitions, identified by developing mechanotransduction research:

1. Mechanical forces of respiration may be converted into chronic oxidative bursts (via NOX and mtROS) through mechanosensors such as Piezo1 and adherens junctions (p120-catenin). This aligns with findings that Piezo1 regulates mechanical force-induced signaling, while NOX-derived ROS drives TME remodeling (Pecchillo Cimmino et al., 2023).
2. The oxidative engine may target the cell’s physical defenses, leading to destruction of the ZO-1/claudin barrier and transient disruption of the lamin A/C nuclear envelope. This is in direct accordance with results demonstrating that ZO-1 depletion decreases junctional mechanical resistance and that oxidative stress leads to breakdown of cellular barriers (Van Itallie et al., 2009).
3. Within a mechanically remodeled chromatin landscape, infiltrating cytoplasmic nucleases (TREX1) and ROS-induced DSBs may create conditions in which the genetic code is rewritten to favor malignant survival (Fang et al., 2023).

This “physical-to-genetic” link may explain why many lung cancers develop in regions of localized fibrosis and mechanical dysfunction, offering a unifying concept for the relationship between tissue physics and genomic instability.

### 7.2 Real-time mechanical monitoring: a technological horizon

Looking ahead, the most important technological advancement will be the transition from static biopsy to real-time *in vivo* mechanical monitoring (Zitnay et al., 2022). The following concepts are presented as theoretical horizons awaiting technological maturation:

Wireless Intra-alveolar Nanosensors: A key goal is the development of biocompatible, wireless nanosensors delivered via inhalation or micro-catheterization into the distal lung (Xu et al., 2021; Lin J. et al., 2024). Such sensors would enable real-time telemetry of the “mechanical load” in the tumor niche, including interstitial fluid pressure, solid stress, and precise magnitude of cyclic load on the alveolar epithelium (Salavati et al., 2022; Khan et al., 2023).

Mechanomic Profiling: When combined with the existing genome and proteome profiles, these may enable creation of a “Mechanomic Map” of the patient’s lung (Dembowitz et al., 2025; Han et al., 2025). This would permit identification of “mechanical hotspots,” localized tissue regions with cyclic strain exceeding threshold levels, before radiologically detectable tumor formation (Zitnay et al., 2022).

### 7.3 Theoretical horizons and future technological frameworks: from personalized physical therapy to mechanomic monitoring

The ideas shared in this section, including wireless nanosensors, mechanomic profiling, and AI-optimized protective ventilation, are introduced as a theoretical guide for the future of mechanopharmacology. All of these concepts are still speculative and have significant technical, biological, and regulatory challenges to overcome before clinical translation. They are not necessarily short-term clinical realities but are rather meant to provoke discussion and hypothesis generation.

The ultimate clinical goal is to develop “personalized mechanotherapy,” which is the modulation of the physical forces themselves as a therapeutic means.

Precision Mechanical Ventilation: AI algorithms could potentially be used to optimize ventilation strategies for patients with high-risk pre-malignant lesions to prevent exposure to pathological stretch thresholds in vulnerable lung segments (Gong et al., 2021). This “protective ventilation” would conceptually deplete the mechanochemical engine of kinetic fuel.

Mechanopharmacology Integration: Mechanopharmacology integration will be personalized using Piezo1 inhibitors and LOX-modulating agents, as discussed in Section 6.2(Nicolas-Boluda et al., 2021; Zhao et al., 2022). Patients with high “stiffness-driven” risk would be treated with intensive LOX-inhibitor therapy, whereas those with high “strain-driven” risk would receive Piezo1-targeted mechanoprotectants.

Future treatments could also involve focused ultrasound or localized vibration therapy to “re-tune” the ECM by physically breaking cross-links in collagen and restoring physiological compliance of the tissue (Lee et al., 2017; Joiner et al., 2020).

### 7.4 Final reflections: a multiscale synthesis

Lung cancer is a complex challenge which needs a multiscale synthesis that goes beyond the disciplinary barriers. The Lung as a Dynamic Physical System starts to bring the vision of the “Mechanochemical Engine” away from the invincible and closer to the quantifiable and interruptible. The next big frontier may be the transition from “Mutation-Centric” oncology to “System-Physics” oncology. This framework offers the potential to not only treat existing malignancies but also, and not least, to prevent the “physical stage” of cancer from ever being triggered, a potential that is in keeping with the new field of mechanotherapeutic research.
